# An Integrated Immune-Related Bioinformatics Analysis in Glioma: Prognostic Signature’s Identification and Multi-Omics Mechanisms’ Exploration

**DOI:** 10.3389/fgene.2022.889629

**Published:** 2022-05-03

**Authors:** Xin Fan, Lingling Zhang, Junwen Huang, Yun Zhong, Yanting Fan, Tong Zhou, Min Lu

**Affiliations:** ^1^ Department of Emergency Medicine, Shangrao Hospital Affiliated to Nanchang University, Shangrao People’s Hospital, Shangrao, China; ^2^ Department of Otolaryngology-Head and Neck Surgery, The First Affiliated Hospital of Nanchang University, Nanchang, China; ^3^ School of Stomatology, Nanchang University, Nanchang, China; ^4^ The First Clinical Medical College of Nanchang University, Nanchang, China; ^5^ Department of Neurosurgery, The Second Affiliated Hospital of Nanchang University, Nanchang, China

**Keywords:** glioma, muti-omics immune-related bioinformatics research, prognostic model, mechanisms’ exploration, tumor immunosuppressive environment, ceRNA regulatory network, IDH1 mutation

## Abstract

As the traditional treatment for glioma, the most common central nervous system malignancy with poor prognosis, the efficacy of high-intensity surgery combined with radiotherapy and chemotherapy is not satisfactory. The development of individualized scientific treatment strategy urgently requires the guidance of signature with clinical predictive value. In this study, five prognosis-related differentially expressed immune-related genes (PR-DE-IRGs) (CCNA2, HMGB2, CASP3, APOBEC3C, and BMP2) highly associated with glioma were identified for a prognostic model through weighted gene co-expression network analysis, univariate Cox and lasso regression. Kaplan-Meier survival curves, receiver operating characteristic curves and other methods have shown that the model has good performance in predicting the glioma patients’ prognosis. Further combined nomogram provided better predictive performance. The signature’s guiding value in clinical treatment has also been verified by multiple analysis results. We also constructed a comprehensive competing endogenous RNA (ceRNA) regulatory network based on the protective factor BMP2 to further explore its potential role in glioma progression. Numerous immune-related biological functions and pathways were enriched in a high-risk population. Further multi-omics integrative analysis revealed a strong correlation between tumor immunosuppressive environment/IDH1 mutation and signature, suggesting that their cooperation plays an important role in glioma progression.

## Introduction

Gliomas are the most common primary intracranial brain tumors, accounting for 81% of malignant brain tumors (Ostrom et al., 2014). As the most malignant and aggressive form of brain tumors, gliomas can cause significant death and morbidity (Ostrom et al., 2014; Ludwig and Kornblum, 2017). Glioma is composed of a variety of malignant cells and non-malignant cells, which can develop in the special environment of the tumor microenvironment (TME), and the tumor evolution of glioma is related to the immune changes in this microenvironment (Wang Q. et al., 2017; Tan et al., 2020). At present, the treatment of glioma is mainly surgery, radiotherapy, chemotherapy, immunotherapy and targeted therapy (Lin et al., 2017; Verburg and de Witt Hamer, 2021).

Intra-tumor heterogeneity, as the main factor affecting the therapeutic effect, has brought enormous scope for the improvement of these therapeutic approaches (Van Meir et al., 2010). It is reported that TME with immunosuppressive properties can help cancer cells evade immune detection, thus leading to cancer progression (Jackson et al., 2011). Studies reveal that TME not only plays a vital role in tumor initiation, progression, and migration, but also affects the generation of therapeutic resistance and malignancy (Huang et al., 2020). TME in human glioblastoma exhibits considerable immune cell infiltration, and the disproportion of immune cells in TME may play an essential role in gliomas (Kong et al., 2020; Qiu et al., 2020). However, due to the strong immunosuppressive microenvironment of gliomas, immunotherapy strategies exhibit a very limited effect on gliomas (Locarno et al., 2020; Xu et al., 2020). Mounting evidence shows that the isocitrate dehydrogenase (IDH) mutation is crucial for the alterations in tumor immunological microenvironment, as indicated by suppression of tumor-infiltrating lymphocytes, natural killer cells and cytotoxic T cells (Bunse et al., 2018; Ren et al., 2019). Moreover, IDH mutations cause neomorphic enzymatic activity that would result in the production of the oncometabolite 2-hydroxyglutarate (2-HG), which can then directly affect the TME (Leca et al., 2021). In gliomas, IDH mutation correlates with decreased PD1/PD-L1 expression (Buege et al., 2018; Mu et al., 2018), and specific inhibitors of mutated IDH may improve the efficacy of immunotherapy in patients with IDH mutated gliomas (Kohanbash et al., 2017). Meanwhile, inhibiting 2-HG production may enhance a host’s ability to immunotherapy response (Leca et al., 2021).

In 2011, Salmena et al. proposed a hypothesis that the crosstalk among messenger RNA (mRNA), transcribed pseudogenes and long non-coding RNA (lncRNA) based on microRNA response elements (MRE) formed a network to regulate RNA transcripts (Salmena et al., 2011). Theoretically, any transcript containing MRE can act as a potential competing endogenous RNA (ceRNA), including mRNAs, lncRNAs, pseudogene RNAs and circular RNAs (circRNAs) (Karreth and Pandolfi, 2013; Qi et al., 2015). CeRNA is reported to be involved in biological processes and plays an important role in disease pathogenesis, such as ovarian cancer (Braga et al., 2020), gastric cancer (Yang et al., 2018), and human colon adenocarcinoma (Zhang et al., 2018). Many lncRNAs play significant regulatory roles in the progression of glioma and can be used as prognostic biomarkers (Langfelder and Horvath, 2008; Liu Z. et al., 2020; Mu et al., 2020; Zhu et al., 2020).

Weighted gene co-expression network analysis (WGCNA) is a new biological method that can describe the connectivity of modules within a comprehensive network and correlate the modules with external sample traits (Langfelder and Horvath, 2008). At present, WGCNA has been successfully applied to the research of numerous cancers, such as breast cancer, non-small-cell lung cancer and ovarian cancer (Niemira et al., 2019; Yin et al., 2020; Su et al., 2021). WGCNA provides an effective way to screen genes that play an important role in tumors. This study aims to screen out prognosis-related differentially expressed immune-related genes (PR-DE-IRGs) that are highly associated with gliomas to construct a prognostic model. Not only should it have excellent prognostic performance, but also abundant clinical application value. Moreover, considering the important role of TME, mutation, cell stemness, ceRNA regulatory network in tumor progression and treatment, we also hope to explore their corresponding potential biological processes in gliomas.

## Materials and Methods

### Collection of Glioma Samples and Identification of Differentially Expressed Immune-Related Genes

**FIGURE 1:**
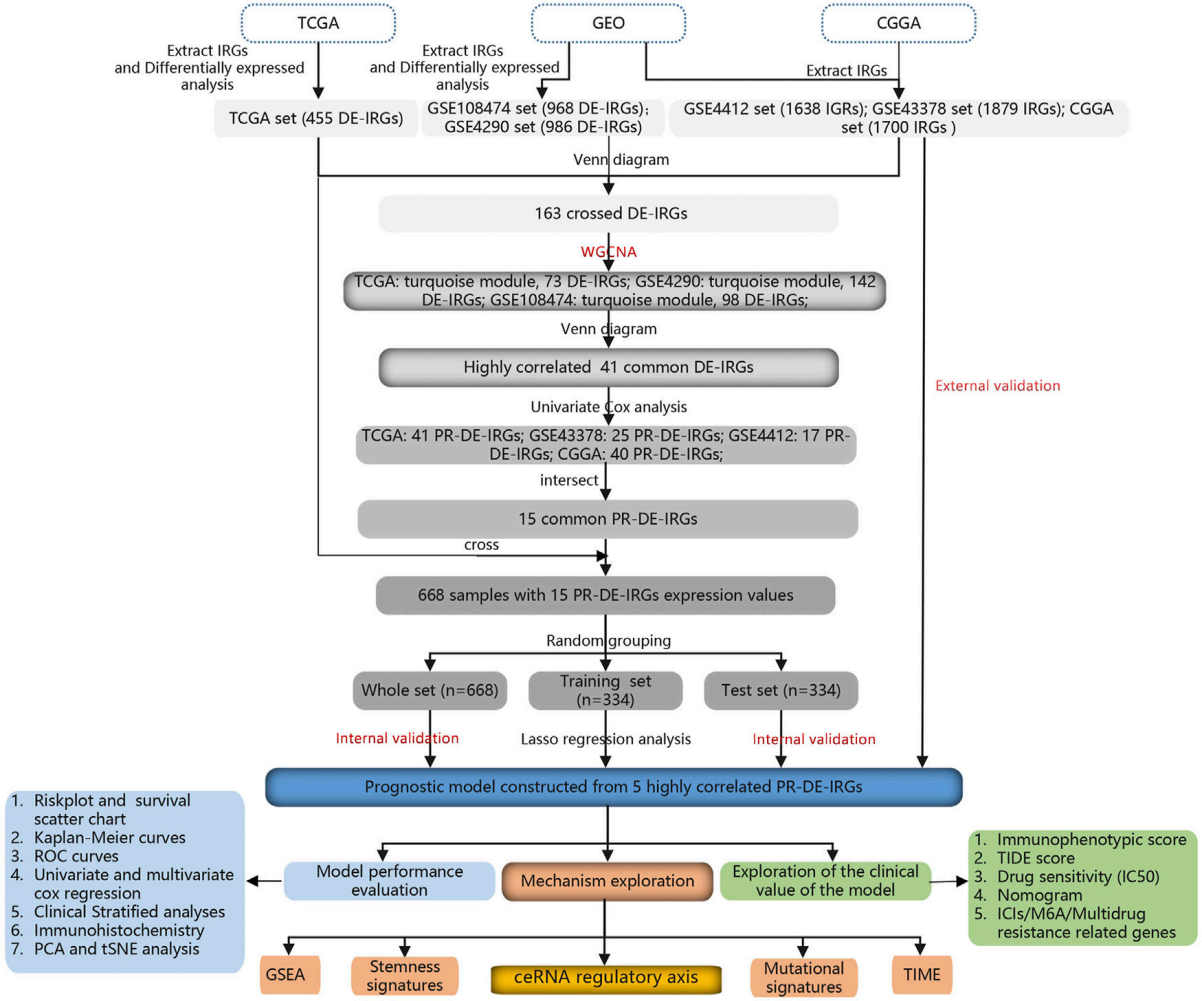
The flowchart of the whole study.

The flowchart shows the RNA sequencing and clinical data sources used in this study (Figure 1): The Cancer Genome Atlas (TCGA; cancergenome.nih.gov/), Gene Expression Omnibus (GEO; ncbi.nlm.nih.gov/geo/) and China Glioma Genome Atlas (CGGA; cgga.org.cn/) databases. While the CGGA database shared the cohort of 1,018 glioma samples, the TCGA database provided the cohort of 703 samples (698 glioma and 5 adjacent normal tissues). The GEO database covered 4 cohorts, including the GSE108474 cohort (148 astrocytoma, 228 glioblastoma multiforme, 67 oligodendroglioma, and 28 normal brain tissues), GSE4290 cohort (26 astrocytoma, 81 glioblastoma, 50 oligodendroglioma, and 23 normal brain tissues), GSE4412 cohort (85 glioma samples) and GSE43378 cohort (50 glioma samples). In addition, the ImmPort (immport.org/home) and InnateDB (innatedb.ca/) databases provided us with a gene list of 6196 immune-related genes (IRGs).

Based on this list, we extracted the RNA sequencing data of 2365, 1879, 1879, 1638, 1879, and 1700 IRGs from the TCGA, GSE108474, GSE4290, GSE4412, GSE43378, and CGGA cohorts, respectively. To identify differentially expressed immune-related genes (DE-IRGs) between tumor and normal tissues from TCGA, we set |log2 fold change| (|log2FC|) >1 and false discovery rate (FDR) < 0.05 as the filter condition. Similarly, FDR <0.05 was used as a new filter condition to identify DE-IRGs from the GSE108474 and GSE4290 cohorts, respectively. Finally, the R package “venn” was used to visualize the overlapping process of DE-IRGs lists and IRGs lists.

### Identification of Differentially Expressed Immune-Related Genes Highly Associated With Glioma Based on Weighted Gene Co-Expression Network Analysis

The RNA sequencing values of 163 common DE-IRGs were extracted from the TCGA cohort to construct the co-expression network among them. After clustering the TCGA samples for eliminating free samples, we used the function pickSoftThreshold to select the best soft power β = 4 to construct the best scale-free network. Based on the formula:

$$a_{ij}=|S_{ij/\beta }$$

(a_ij_: adjacency matrix between gene i and gene j, S_ij_: similarity matrix which is done by Pearson correlation of all gene pairs, *β*: softpower value), we created an adjacency matrix, and converted it to a topological overlap matrix (TOM) and a corresponding dissimilarity (1-TOM) (Yip and Horvath, 2007). Then, we took 1-TOM as the distance to cluster the genes, and built a dynamic pruning tree to identify the gene modules (Yip and Horvath, 2007). Finally, after merging similar modules with 75% similarity, we identified 3 modules. Likewise, we chose the best soft power *β* = 4 and *β* = 5 to identify two modules from the GSE108474 and GSE4290 cohorts, respectively. The turquoise modules of the GSE4290, GSE108474, and TCGA cohorts all exhibited the strongest positive correlation with tumor status. The genes of these three modules were extracted separately to obtain the common DE-IRGs highly associated with glioma.

### Acquisition of Prognosis-Related Differentially Expressed Immune-Related Genes and Model Construction

Samples with complete overall survival (OS) and RNA sequencing data from 6 cohorts were extracted for subsequent analysis, respectively. To obtain corresponding PR-DE-IRGs in the TCGA, GSE4412, GSE43378 and CGGA cohorts, we performed univariate Cox analysis with a cutoff value of *p* < 0.05. At the same time, we also performed Kaplan-Meier survival analysis on the common PR-DE-IRGs in these four cohorts to explore the relationship between their expression and OS.

668 samples from TCGA cohort were randomly matched to the training set (*n* = 334) and test set (*n* = 334) on average. Lasso regression analysis can screen out highly relevant PR-DE-IRGs from the training set, thereby minimizing the risk of overfitting the screening features, and achieving the purpose of accurately predicting the patients’ prognosis (Lu et al., 2022). The optimal penalty parameter (*λ*) obtained by the minimum 10-fold cross validation was used to determine five PR-DE-IRGs and corresponding coefficients for constructing the prognostic model. We calculated each sample’s risk score from four cohorts by the formula:

Risk score=∑(PR−DE−IRGs expression values×corresponding coefficient)

### Verification of Model’s Predictive Ability

CCGA set, GEO’s GSE4412 and GSE43378 sets, and TGGA’s training, test and whole sets were used for the verification of model’s predictive ability. We used the median risk score of samples from each cohort as a cutoff point to classify samples into high-risk and low-risk groups. Kaplan-Meier survival analysis was used to validate the model’s ability in differentiating the glioma patients’ prognosis. We also plotted the receiver operating characteristic (ROC) curves to evaluate the accuracy of the model in predicting prognosis. Univariate and multivariate Cox regression analyses further identified the role of risk score as an independent predictor of prognosis. In addition, we also performed principal component analysis (PCA) and t-distributed random neighborhood embedding (t-SNE) to evaluate the model’s ability to discretize samples through the R package “Rtsne".

### Gene Set Enrichment Analysis

To enrich for potential biological functions and pathways involved in different risk groups, we ran Gene Set Enrichment Analysis (GSEA) based on the R package “clusterProfiler”. In this process, we used “c5.go.v7.4.symbols.gmt” and “c2.cp.kegg.v7.4.symbols.gmt” as reference gene sets and set nominal *p* value < 0.05 as the filter condition.

### Tumor Immunosuppressive Environment and Immune Infiltration Type Analyses

First, the overall stromal and immune cell scores for each TCGA sample were calculated using the ESTIMATE algorithm (sourceforge.net/projects/estimateproject/). We compared their differences between different risk groups, and analyzed their correlations with risk score through Spearman. Next, based on the single-sample gene set enrichment analysis (ssGSEA) of the R packages “GSEABase” and “gsva”, we quantified the scores of 16 immune cells and 13 immune functions in each sample. After visualizing their distribution across all samples using a heatmap, similar differential expression and correlation analyses were again applied to explore their relationship with the model.

Six types of immune infiltration were identified in human tumors, which corresponded from tumor promoting to tumor suppressing respectively, namely C1 (wound healing), C2 (INF-g dominant), C3 (inflammatory), C4 (lymphocyte depleted), C5 (immunologically quiet), and C6 (TGF-b dominant) (Lin et al., 2021). The two-way ANOVA was used to explore association between risk score and different types of immune infiltration. To compare the distribution of C3, C4, and C5 subtypes between different risk groups, we ran a chi-square test.

### Cell Stemness Analysis of Glioma

The one-class logistic regression machine learning algorithm (OCLR) provided targeted training for stem cells (embryonic stem cells; induced pluripotent stem cells) and their differentiated ectoderm, mesoderm and endoderm progenitor cells (Malta et al., 2018). Malta et al. calculated stemness index (mDNAsi) for each TCGA sample by OCLR, ranging from low (zero) to high (one) (Malta et al., 2018). The mDNAsi data from 564 glioma samples from this study were used for our analysis. Next, we explored the mDNAsi difference between different risk groups/clinical subgroups as well as the correlation between mDNAsi and risk score.

### Mutation Analysis

After counting the somatic gene mutation data of gliomas from TCGA, we obtained the top 30 genes with the highest mutation frequency. The R package “GenVisR” was used to visualize the statistical results of these 30 genes’ mutation in different risk groups. A similar method was applied to the mutated DE-IRGs highly associated with glioma. While analyzing the association between tumor mutation burden (TMB) and model, we also explored the effect of TMB on prognosis. Based on the mutation status of IDH1, the samples were divided into wild and mutant groups. In addition to the differences in the expression of 5 PR-DE-IRGs and CD274 between the two groups, the differences in the risk score, survival probability, 16 immune cells and 13 immune functions were compared.

### Construction of a Comprehensive Regulatory Network Composed of Interconnected ceRNAs

To further explore the possible mechanisms involved in glioma progression from the perspective of ceRNA, we selected BMP2 as the target gene to construct a comprehensive regulatory network composed of interconnected ceRNAs. After annotating the miRNA expression data from TCGA using mature miRNA file from mirbase database (mirbase.org/), we obtained a miRNA expression matrix for 535 samples (530 glioma and 5 adjacent normal tissues). The starBase database (starbase.sysu.edu.cn/) contains multiple target gene prediction programs (PITA, RNA22, miRmap, microT, miRanda, PicTar, and TargetScan). We selected miRNAs that appeared more than 2 times in all prediction programs as candidate miRNAs for BMP2. During this period, Cytoscape (v3.7.2) was used to map the co-expression network of all predicted miRNAs and BMP2. Based on the correlation analyses between these miRNAs and BMP2 expression (filter condition: correlation coefficient < −0.4 and *p* < 0.001), we further screened out the differentially expressed miRNAs between glioma and normal tissues (filter condition: |log2FC| >1 and *p* < 0.05). Ultimately, only 3 miRNAs (hsa-miR-365a-3p, hsa-let-7e-5p and hsa-miR-98-5p) showed significant survival differences in the further Kaplan-Meier survival analyses (filter condition: *p* < 0.05).

Next, starBase was used to predict candidate lncRNAs that may bind to hsa-miR-365a-3p, hsa-let-7e-5p and hsa-miR-98-5p, respectively. Similarly, the correlation analyses between these lncRNAs and hsa-miR-365a-3p/hsa-let-7e-5p/hsa-miR-98-5p expression (filter condition: correlation coefficient < −0.2 and *p* < 0.001), as well as their difference analyses between glioma and normal tissues (filter condition: *p* < 0.05) were run separately. Next, 13/13/11 lncRNAs that might bind to hsa-miR-365a-3p/hsa-let-7e-5p/hsa-miR-98-5p were used for further screening. Cytoscape was run again to map the comprehensive regulatory network composed of interconnected ceRNAs. Finally, 4/2/4 lncRNAs upstream of hsa-miR-365a-3p/hsa-let-7e-5p/hsa-miR-98-5p were identified from the following three analysis steps: 1) The correlation analysis between 13/13/11 lncRNAs and hsa-miR-365a-3p/hsa-let-7e-5p/hsa-miR-98-5p expression (filter condition: coefficient < −0.28 and *p* < 0.001); 2) The correlation analysis between 13/13/11 lncRNAs and BMP2 expression (filter condition: correlation coefficient < −0.4 and *p* < 0.001); 3) The differential analysis of 13/13/11 lncRNAs expression between glioma and normal tissues (filter condition: |log2FC| >1 and *p* < 0.05). Kaplan-Meier survival analysis was again used to explore the influence of these lncRNAs on prognosis. We highlighted these lncRNAs with yellow in the comprehensive regulatory network.

To further predict the regulatory network constructed by circRNAs, miRNAs, and BMP2, we also downloaded the circRNAs expression data of the GSE165926 set (12 gliomas and 4 normal brain tissues) and the miRNAs expression data of the GSE138764 set (33 astrocytoma and 9 normal brain tissues). Based on the TCGA and GSE138764 sets, respectively, the differentially expressed miRNAs (DE-miRNAs) between glioma and normal brain tissues were obtained (filter condition: |log2FC| >2 and *p* < 0.05). Prognosis related differentially expressed miRNAs (PR-DE-miRNAs) were obtained by Kaplan-Meier survival analysis based on TCGA data (filter condition: *p* < 0.001). We re-identified the common miRNAs (hsa-miR-129-5p and hsa-miR-381-3p) from the PR-DE-miRNAs of the TCGA set, the DE-miRNAs of the GSE138764 set, and the miRNAs upstream of BMP2 predicted by starBase as candidate miRNAs that may regulate BMP2 expression. StarBase was again used to predict circRNAs that might bind to hsa-miR-129-5p and hsa-miR-381-3p, respectively. A similar approach was used to obtain differentially expressed circRNAs (DE-circRNAs) from the GSE165926 set. We got the common circRNAs from DE-circRNAs of the GSE165926 set and the circRNAs predicted by starBase as candidate circRNAs that might bind to hsa-miR-129-5p/hsa-miR-381-3p, respectively. Finally, only the up-regulated hsa_circ_0004662 and hsa_circ_0007548 in gliomas upstream of hsa-miR-129-5p satisfy the regulation of ceRNA. Cytoscape plots the corresponding regulatory network.

### Clinical Application of Prognostic Model

We obtained the immunophenotypic score (IPS) of glioma patient from the Cancer Immunology Atlas (TCIA) database (tcia.at/home). Previous studies showed that immunogenicity increases with increasing IPS score (Liu et al., 2020b). By analyzing the gene expression of the four cell types that determine immunogenicity (effector cells, immunosuppressive cells, major histocompatibility complex molecules and immunomodulators), the IPS of the sample was obtained (Liu et al., 2020b). Spearman correlation analysis was used to analyze the correlation between 4 types of IPSs and 5 PR-DE-IRGs expression/risk score. We used the violin plot to show the difference of Tumor Immune Dysfunction and Exclusion (TIDE), microsatellite instability (MSI), T cell dysfunction and exclusion score between high and low risk groups. TIDE scores were calculated online at the TIDE website (tide.dfci.harvard.edu/).

We accessed the NCI-60 database containing 60 different cancer cell lines from 9 different types of tumors through the CellMiner interface (discover.nci.nih.gov/cellminer). The Pearson correlation analysis was run to explore the relationship between 5 PR-DE-IRGs expression and 263 drugs approved by the food and drug administration or clinical trials.

### Establishment and Verification of a Combined Nomogram

We used the R package “regplot” to combine the risk score with two clinical prognostic factors (grade, age) to establish a combined nomogram, which can more accurately predict the survival probability of glioma patients at 1-, 2-, and 3- years. To better prove the accuracy and effectiveness of nomogram, we constructed internal calibration curves and ROC curves based on the training, test, and whole sets.

### Immune Checkpoint Inhibitors/N6-Methyladenine/Multidrug-Resistance Related Gene Expression Analysis

As the most common covalent modification of RNA at the posttranscriptional level, N6-methyladenine (m6A) mRNA modification plays a key role in gliomas through various mechanisms and associates with clinicopathological features and prognosis of gliomas patients, showing a great clinical significance (Zhang et al., 2020; Zhang et al., 2021). To explore the correlation between risk score and Immune Checkpoint Inhibitors (ICIs)/m6A related gene expression, we again used Spearman correlation analysis. In addition, we also compared the expression differences of ICIs/m6A related genes between high and low risk groups. Studies have shown that the multidrug resistance-associated protein (MRP or ABCC) expression may be related to the inherent multidrug resistance in human gliomas (Mohri et al., 2000), so we respectively analyzed the correlation between the ABCC1/ABCC3 expression and the risk score, as well as their expression differences between different risk groups.

### Stratified Analyses and Immunohistochemical Staining Images Verification

We used a heatmap to show the distribution of clinical characteristics across all TCGA samples from different risk groups. To visualize the differences in risk score between different subgroups for each clinical characteristic, we drew boxplots. Kaplan-Meier survival analysis was used to evaluate the model’s predictive ability in each subgroup with different clinical characteristics. We obtained the immunohistochemical (IHC) staining results from the human protein atlas database (proteinatlas.org/) to verify the differences in protein levels of 5 modelled genes between normal tissues and glioma tissues. Finally, only 4 PR-DE-IRGs (HMGB2, CCNA2, CASP3, and APOBEC3C) IHC staining images were obtained.

### Statistical Method

We used Student’s t-test or Wilcoxon signed-rank test to compare the differences between continuous variables, while the differences between categorical variables were compared by Chi-square test or Fisher’s exact test (Fan et al., 2021). Univariate cox regression analysis was used to select PR-DE-IRGs. Lasso and multiple Cox regression were used to construct prognostic model and nomogram. Univariate and multivariate Cox regression analyses were also used to identify independent prognostic factors. Spearman or Pearson correlation analyses were used to analyze the correlation among variables. The above analyses were run in the R software (version 4.0.3), Perl and SPSS Statistics 22.

## Results

### Extraction of Common Differentially Expressed Immune-Related Genes

**FIGURE 2:**
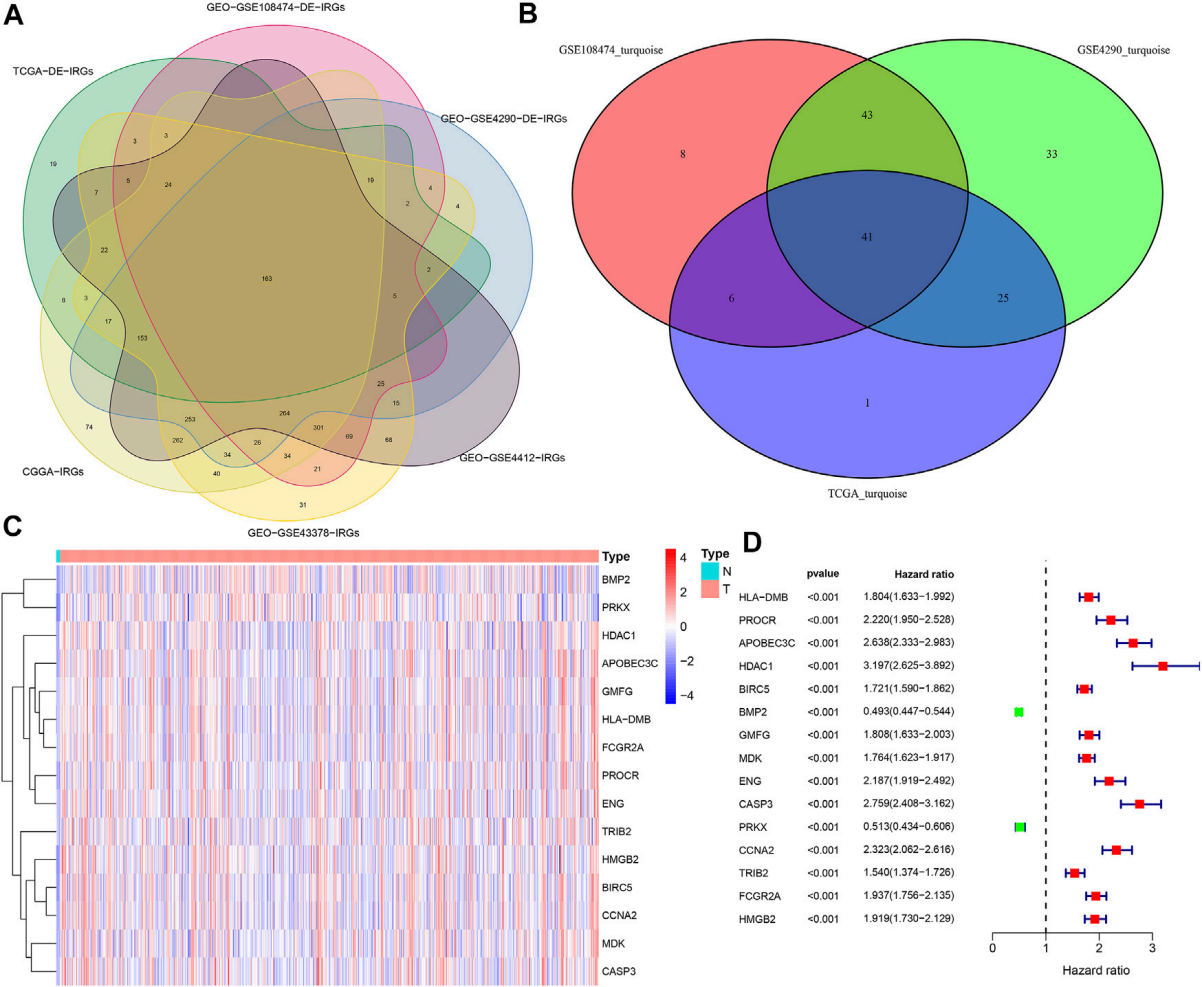
Extraction of PR-DE-IRGs. **(A)** Venn diagram showing the overlapping process of DE-IRGs lists from three sets and IRGs lists from three other sets. **(B)** Venn diagram showing the overlapping process of genes from TCGA turquoise module, GSE4290 turquoise module and GSE108474 turquoise module. **(C)** Heatmap reflecting the expression levels of 15 PR-DE-IRGs in TCGA samples. **(D)** Forest plot showing the univariate Cox regression analysis results of 15 PR-DE-IRGs in TCGA.

We extracted 455 DE-IRGs (189 genes were down-regulated and 266 genes were up-regulated in glioma) from the TCGA cohort (Supplementary Figure S1) and 968 DE-IRGs (492 genes were up-regulated and 476 genes were down-regulated in glioma) from the GSE108474 cohort (Supplementary Figure S2). In the same way, we also identified 986 DE-IRGs from the GSE4290 cohort (Supplementary Figure S3). Finally, by Venn diagram based on the 6 cohorts, we obtained 163 common DE-IRGs (Figure 2A).

### Identification of Differentially Expressed Immune-Related Genes Highly Associated With Glioma Based on Weighted Gene Co-Expression Network Analysis

**FIGURE 3:**
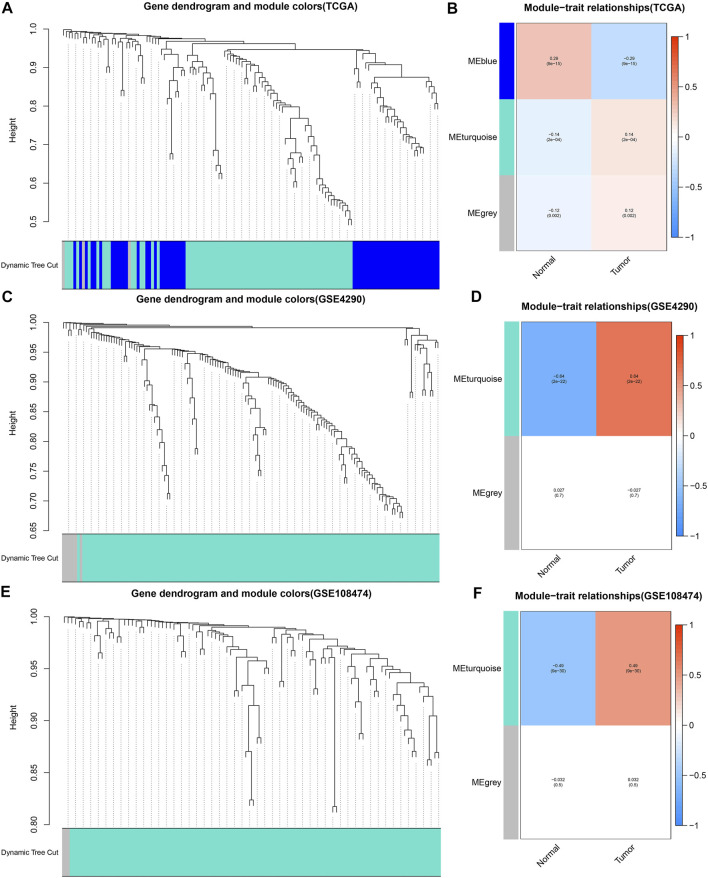
Merged dynamic pruning trees and heatmaps of TCGA, GSE4290, and GSE108474 cohorts. **(A**,**C**,**E)** Dynamic pruning trees merging similar gene modules into one gene module. **(B**,**D**,**F)** Heatmaps showing correlation between gene modules and tumor status. The color label on the left is followed by the corresponding module. The two squares after the gene modules shows the correlation coefficients and *p* values between the gene modules and normal/tumor status, respectively.

The merged dynamic pruning tree in Figures 3A,C,E showed the results of genes and corresponding modules matched in the TCGA, GSE4290 and GSE108474 cohorts, respectively. Then we identified 3 modules from the TCGA cohort (Figure 3B), 2 modules from the GSE4290 cohort (Figure 3D), and 2 modules from the GSE108474 cohort (Figure 3F). The heatmaps (Figures 3B,D,F), reflecting the correlation between modules and tumor status, showed that the turquoise module from the GSE4290 cohort had the strongest correlation with tumor status (|correlation coefficient| = 0.64, *p* < 0.001, Figure 3D). Finally, we got 41 common DE-IRGs highly associated with glioma using the Venn diagram (Figure 2B).

### Construction of Prognostic Model Based on Prognosis-Related Differentially Expressed Immune-Related Genes

**FIGURE 4:**
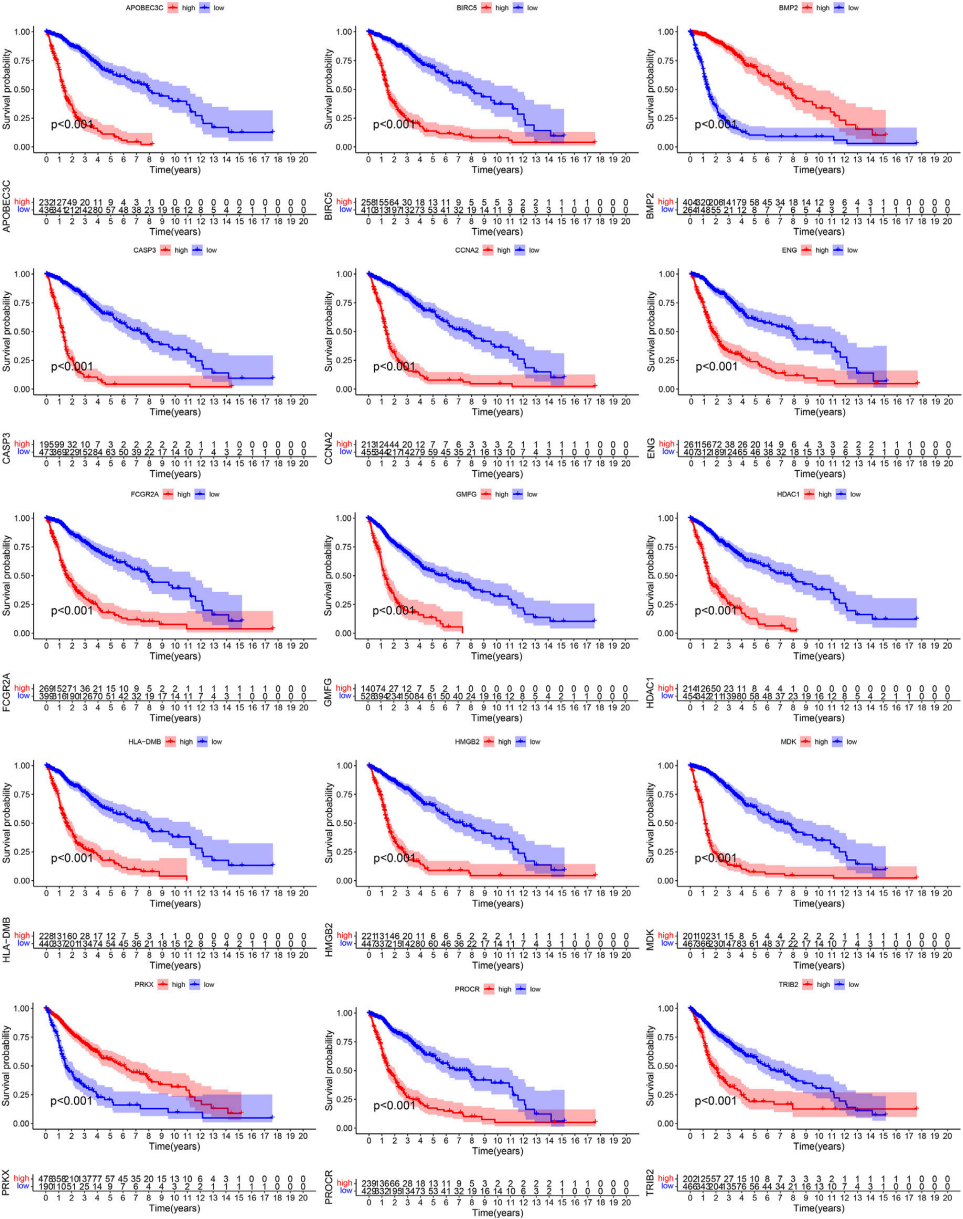
Survival curves of 15 PR-DE-IRGs from TCGA cohort.

668 samples from the TCGA cohort, 85 samples from the GSE4412 cohort, 50 samples from the GSE43378 cohort, and 983 samples from the CGGA cohort were used in the subsequent analyses. Supplementary Tables S1,2 showed the statistical results of these samples’ clinical characteristics. During the overlapping process of TCGA cohort’s 41 PR-DE-IRGs, GSE4412 cohort’s 17 PR-DE-IRGs, GSE43378 cohort’s 25 PR-DE-IRGs, and CGGA cohort’s 40 PR-DE-IRGs, we obtained 15 common PR-DE-IRGs (Supplementary Figure S4A). The forest plots showed the univariate Cox analyses’ results of 15 common PR-DE-IRGs from 4 cohorts (TCGA cohort: Figure 2D; CGGA cohort: Supplementary Figure S4B; GSE4412 cohort: Supplementary Figure S4C; GSE43378 Cohort: Supplementary Figure S4D). Except for BMP2 and PRKX (HR < 1), the remaining 13 genes (HR > 1) were identified as prognostic risk factors in all cohorts. This conclusion was supported by further Kaplan-Meier survival analysis results (TCGA cohort: Figure 4; CGGA cohort: Supplementary Figure S5; GSE4412 cohort: Supplementary Figure S6; GSE43378 Cohort: Supplementary Figure S7). In addition, we also used a heatmap to visualize the expression levels of these 15 PR-DE-IRGs in the TCGA cohort (Figure 2C).

Finally, lasso regression analysis determined 5 PR-DE-IRGs and corresponding coefficients (Supplementary Table S3) to build the model according to the optimal penalty parameter (*λ*) (Supplementary Figures S8A,B).

### Verification of Model’s Predictive Ability

**FIGURE 5:**
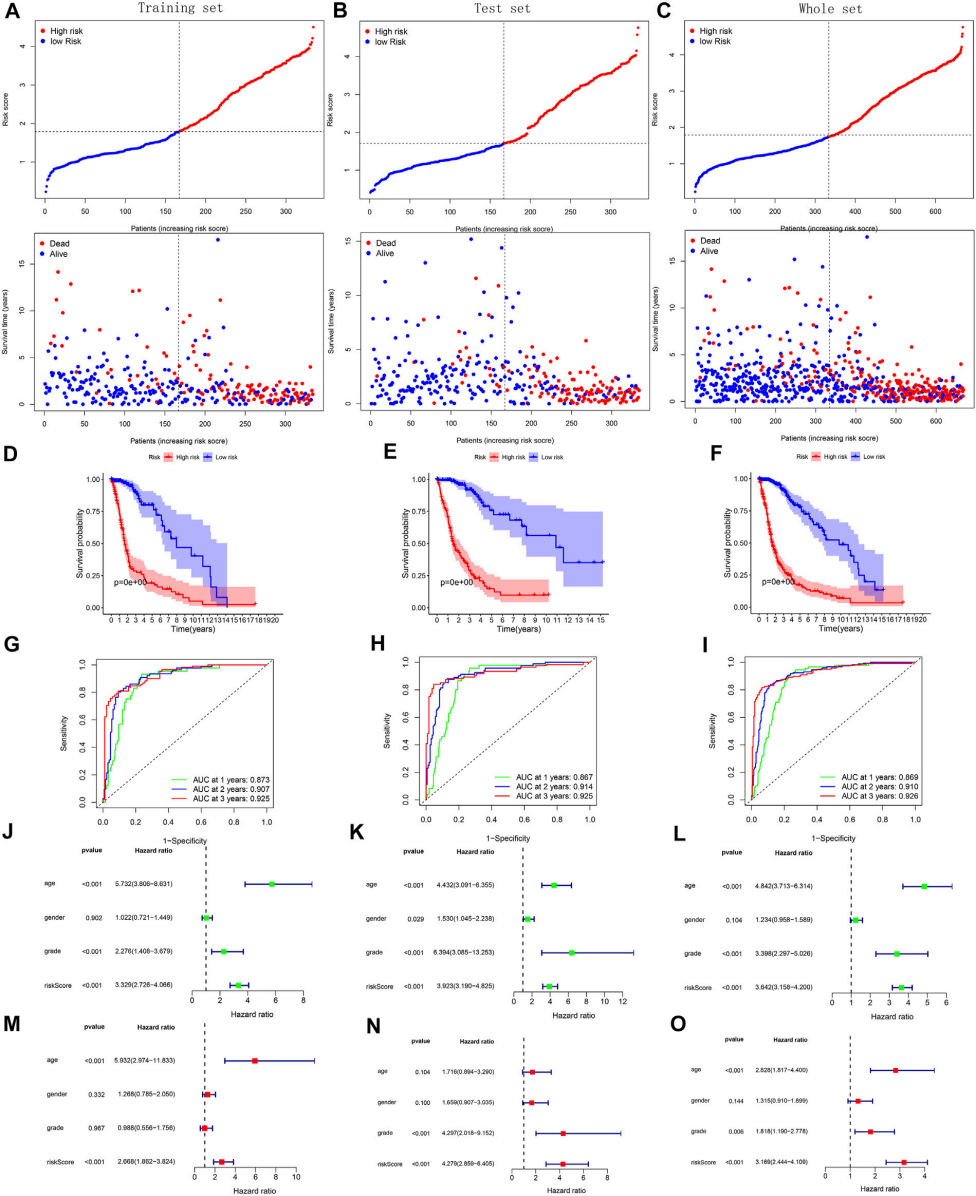
Validation of model’s predictive performance using the data of the training, test, and whole set from TCGA. **(A**–**C)** Risk curves and survival point plots with increasing risk score. **(D**–**F)** Survival curves. **(G**–**I)** ROC curves of OS at 1-, 2-, 3-years. AUC >0.5 is considered to have a predictive value. **(J**–**O)** Forest plots reflecting the results of univariate and multivariate cox regression analyses.

**FIGURE 6:**
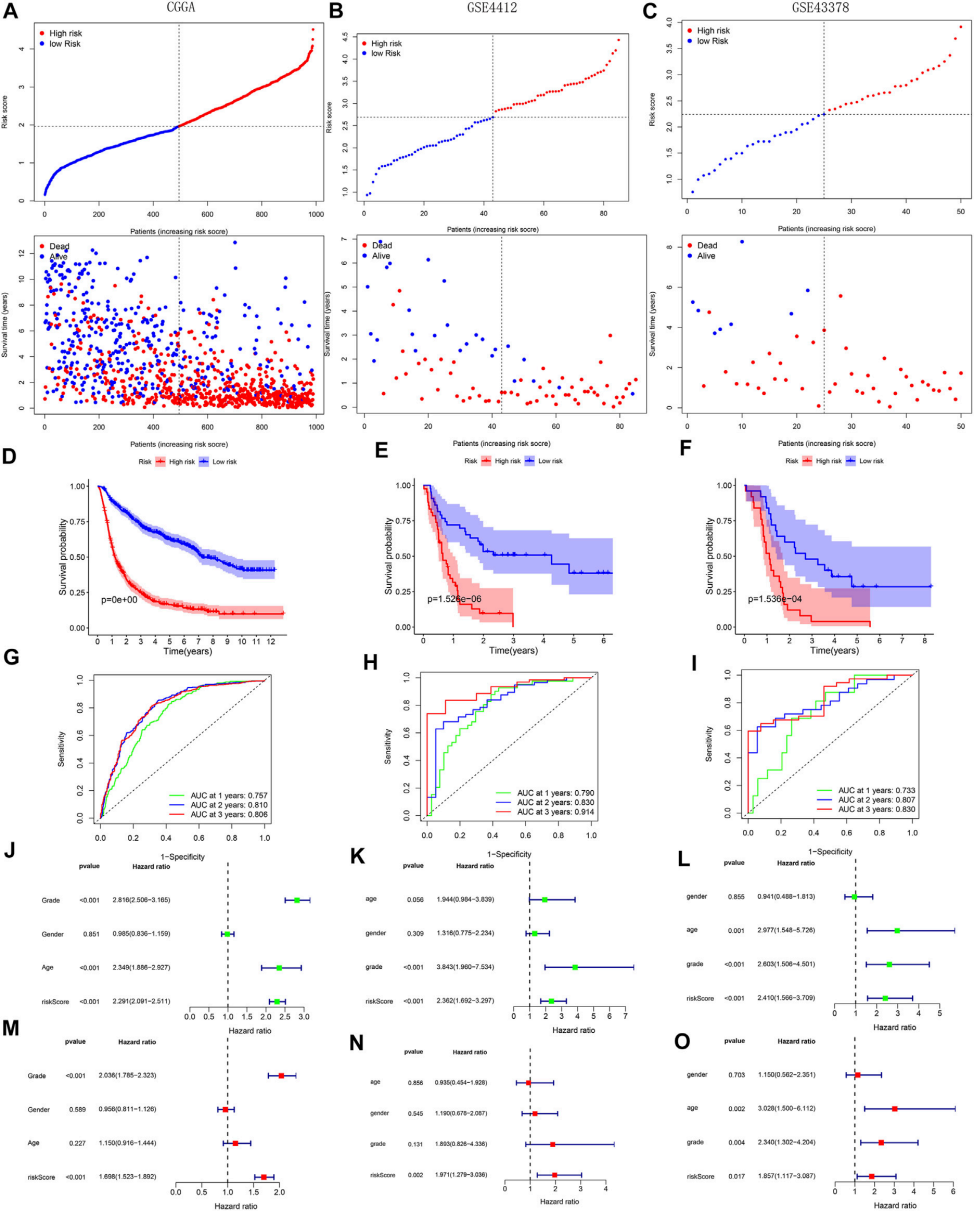
Validation of model’s predictive performance using the data of the CGGA, GSE4412, and GSE43378 cohorts. **(A**–**C)** Risk curves and survival point plots with increasing risk score. **(D**–**F)** Survival curves. **(G**–**I)** ROC curves of OS at 1-, 2-, 3-years. AUC >0.5 is considered to have a predictive value. **(J**–**O)** Forest plots reflecting the results of univariate and multivariate cox regression analyses.

With survival point plots, we observed more deceased samples in the high-risk group (Figures 5A–C and Figures 6A–C). Kaplan-Meier survival curves further showed the lower survival probability for high-risk group samples (Figures 5D–F and Figures 6D–F). To better verify the model’s predictive performance, we drew the ROC curves. In the ROC curves of three TCGA’s sets, all area under curve (AUC) values were greater than 0.85 (Figures 5G–I), showing our model’s excellent performance. This conclusion was also supported by the ROC curves of the three external cohorts (all AUC >0.7) (Figures 6G–I). The risk score was identified as a prognostic risk factor by the univariate Cox regression analysis (Figures 5J–L and Figures 6J–L). After adjusting for various clinical factors, the multivariate Cox regression analysis further determined the risk score as an independent prognostic risk factor (Figures 5M–O and Figures 6M–O). Finally, both PCA and t-SNE dot plots showed that the risk grouping separated the samples well (Supplementary Figure S9).

### Gene Set Enrichment Analysis Enrichment Analysis and Tumor Immunosuppressive Environment

**FIGURE 7:**
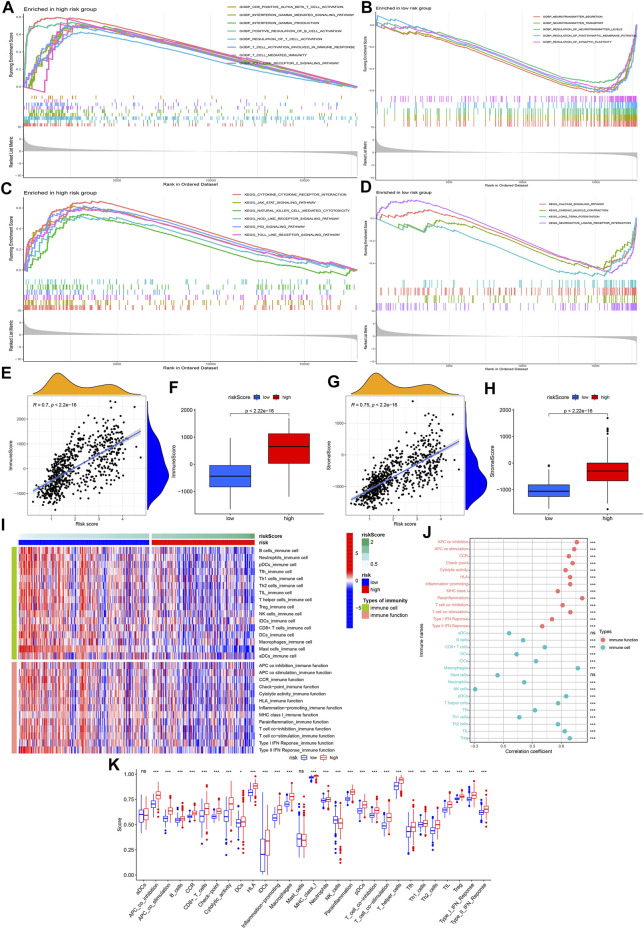
GSEA and tumor immunosuppressive environment analysis. **(A**,**B)** GO enrichment analysis results of high-risk group and low-risk group. **(C**,**D)** KEGG enrichment analysis results of high-risk group and low-risk group. **(E**,**G)** Correlation analysis results between risk score and overall immune score/stromal score. **(F**,**H)** Difference analysis results of overall immune score/stromal score between the high-risk and low-risk groups. **(I)** Heatmap visualizing 16 immune cell and 13 immune function scores for each sample. **(J)** Correlation analysis results between risk score and 16 immune cells/13 immune functions. **(K)** Difference analysis results of 16 immune cells/13 immune functions between the high-risk and low-risk groups. The symbols on the right and top of the graph represent different *p* values, respectively. ns: no significance; *p < 0.05; **p < 0.01; ***p < 0.001.

In the low-risk group, we found GO analysis results (neurotransmitter secretion, neurotransmitter transport, regulation of neurotransmitter levels, regulation of postsynaptic membrane potential, and regulation of synaptic plasticity) (Figure 7B) and KEGG analysis results (calcium signaling pathway, cardiac muscle contraction, long-term potentiation, and neuroactive ligand-receptor interaction) (Figure 7D) were all closely related to the nervous system. In the high risk group, both GO analysis results (CD8-positive alpha-beta T cell activation, interferon gamma mediated signaling pathway, interferon gamma production, positive regulation of B cell activation, regulation of T cell activation, T cell activation involved in immune response, T cell mediated immunity and toll-like receptor 2 signaling pathway) (Figure 7A) and KEGG analysis results (cytokine-cytokine receptor interaction, JAK-STAT signaling pathway, natural killer cell mediated cytotoxicity, NOD-like receptor signaling pathway, p53 signaling pathway and toll-like receptor signaling pathway) (Figure 7C) contained numerous immune-related biological processes. Because GSEA exhibited a strong link between high-risk group and immunity, we further analyzed the relationship between the model and the immune microenvironment. Figures 7E,F showed a positive correlation between risk score and overall immune cell score, and higher overall immune cell score in the high-risk group. Similar results were observed in the overall stromal cell score (Figures 7G,H). After visualizing 16 immune cells and 13 immune functions scores for each sample (Figure 7I), we further analyzed their correlation with risk score, and their differences between high-risk and low-risk groups. The results showed that the remaining immune cells and functions scores were positively correlated with risk score except for natural killer (NK) and mast cells (Figure 7J). The above results were consistent with the differential analysis results presented in Figure 7K.

### Immune Infiltration Type and Cell Stemness Analyses

**FIGURE 8:**
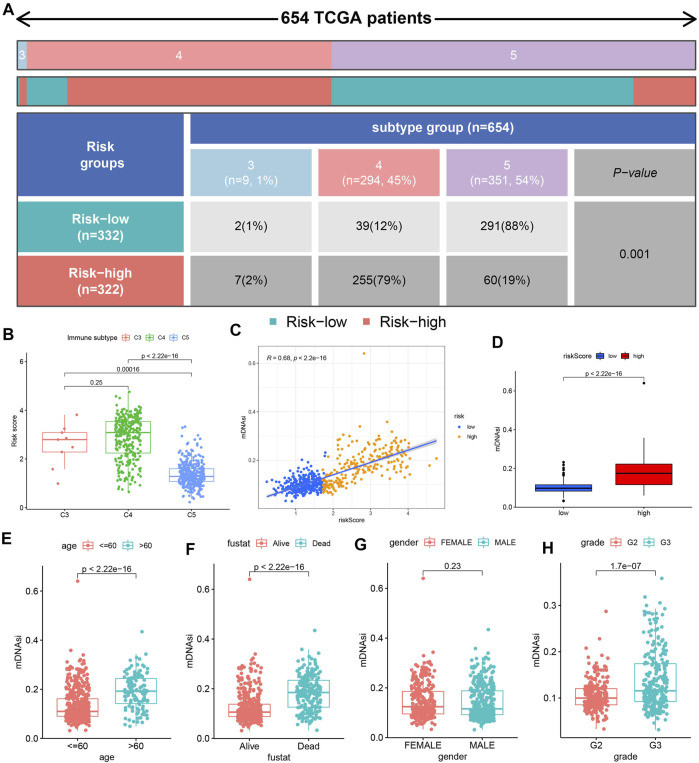
Immune infiltration type and cell stemness analyses. **(A)** Table showing the distribution of immune infiltrating subtypes (C3, C4, and C5) between the different risk groups. **(B)** Risk score difference among the 3 immune infiltrating subtypes. **(C)** Correlation analysis results between mDNAsi and risk score. **(D)** Difference analysis results of mDNAsi between high-risk and low-risk groups. **(E**–**H)** Difference analysis results of mDNAsi between different clinical feature subgroups.

Figure 8A showed the distribution of C3, C4, and C5 between the high-risk and low-risk groups. We observed the most C5 subtypes in the low-risk group and the most C4 subtypes in the high-risk group. The results in Figure 8B further verified the above situation. Cell stemness analysis was of great significance to the epigenetic characteristics of glioma patients. We observed a significant correlation between the mDNAsi and risk score (Figure 8C), and higher mDNAsi in the high-risk group (Figure 8D). Finally, we observed higher mDNAsi in the age >60 group (Figure 8E), deceased group (Figure 8F) and G3 group (Figure 8H). But, there was no significant difference in mDNAsi between different gender subgroups (Figure 8G).

### Mutation Analysis

**FIGURE 9:**
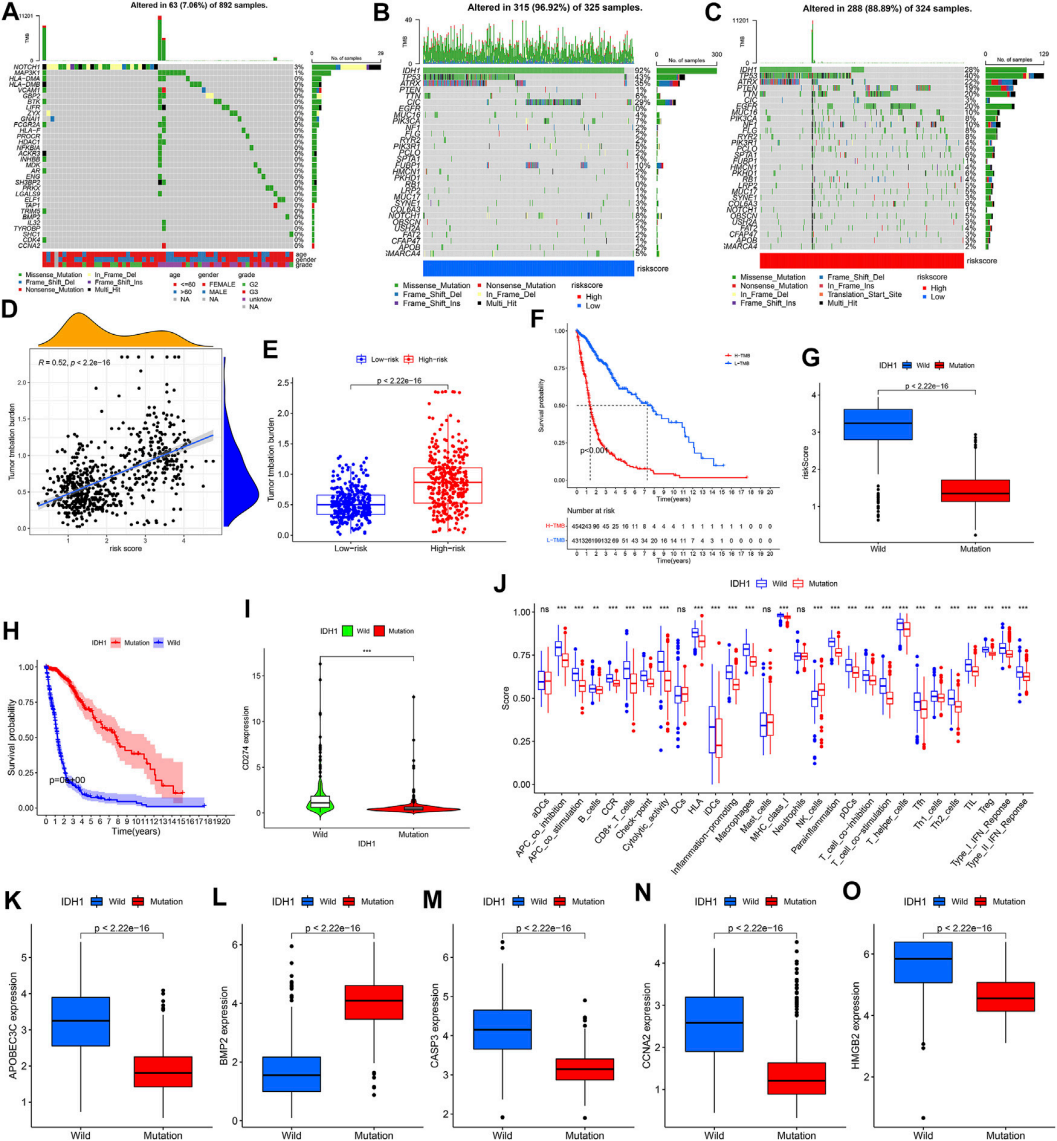
Mutation analysis. **(A)** Waterfall plot reflecting the statistical results of mutations in 32 mutated DE-IRGs highly associated with gliomas. **(B**,**C)** Waterfall plots visualizing the statistical results of these 30 genes’ mutation in different risk groups. The right panel of the waterfall plot showing the mutation frequency. The different colors at the bottom of the waterfall plot showing different mutation types and clinical characteristics types. The histogram above the waterfall plot showing the TMB statistical results for each sample. **(D)** Correlation analysis results between risk score and TMB. **(E)** TMB difference between the high-risk and low-risk groups. **(F)** Difference in survival probability between high and low TMB groups. **(G)** Risk score difference between IDH1 mutant and wild-type group. **(H)** Difference in survival probability between IDH1 mutant and wild-type groups. **(I**,**K**–**O)** Expression difference of CD274/APOBEC3C/BMP2/CASP3/CCNA2/HMGB2 between IDH1 mutant and wild-type groups, respectively. **(J)** Difference in 16 immune cells and 13 immune functions between the IDH1 mutant and wild-type groups.

From Figure 9A, we found that 32 of the 41 DE-IRGs highly associated with glioma were mutated. The mutation frequency of IDH1 was the highest in different risk groups (Figures 9B,C). We observed that TMB was positively correlated with risk score, which was supported by further differential analysis (Figures 9D,E). The survival analysis in Figure 9F showed that the high TMB group samples have significantly lower survival probabilities (*p* < 0.001). Lower risk score and higher survival probability were observed in the IDH1 mutant group (Figures 9G,H). Except for BMP2, the expression levels of other genes (CD274, APOBEC3C, CASP3, CCNA2 and HMGB) in the wild-type group were higher (Figures 9I,K–O). Figure 9J showed that NK cells infiltrated more abundantly in the mutant group, while the others infiltrated more abundantly in the wild-type group.

### Construction of a Comprehensive Regulatory Network Composed of Interconnected ceRNAs

**FIGURE 10:**
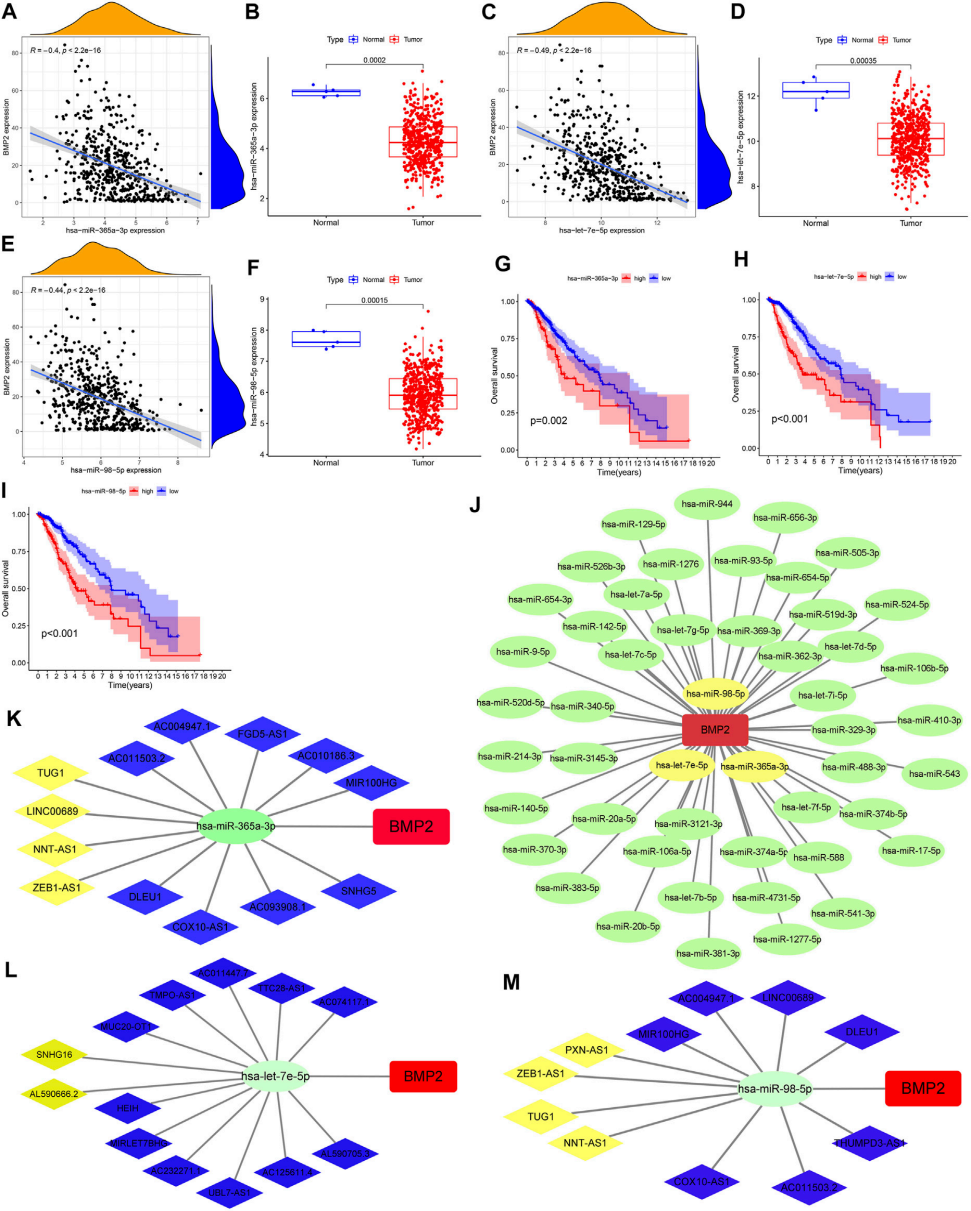
Construction of a comprehensive regulatory network composed of interconnected ceRNAs. **(A**,**C**,**E)** Correlation analysis results between BMP2 and hsa-miR-365a-3p/hsa-let-7e-5p/hsa-miR-98-5p. **(B**,**D**,**F)** Difference analysis results of miR-365a-3p/let-7e-5p/miR-98-5p between the glioma and the normal groups. **(G**–**I)** Kaplan-Meier survival curves of hsa-miR-365a-3p/hsa-let-7e-5p/hsa-miR-98-5p. **(J)** Regulatory network between 52 predicted miRNAs and BMP2. **(K**–**M)** CeRNA regulatory networks based on lncRNAs, hsa-miR-365a-3p/hsa-let-7e-5p/hsa-miR-98-5p and BMP2.

After reasonable prediction, we obtained 52 upstream miRNAs that may bind to BMP2. Figure 10J showed the regulatory network between these 52 miRNAs and BMP2. Supplementary Table S4 showed all predicted miRNAs’ correlation and differential analysis results. Figures 10A–I visualized the results of the correlation, difference and Kaplan-Meier survival analyses for 3 miRNAs (hsa-miR-365a-3p, hsa-let-7e-5p, and hsa-miR-98-5p). Therefore, these three miRNAs may become the most potential miRNAs regulating BMP2 expression in glioma.

Based on the starBase database, we successfully obtained 112/101/112 possible upstream lncRNAs of miR-365a-3p/let-7e-5p/miR-98-5p, respectively. Figures 10K–M showed the comprehensive regulatory network composed of interconnected ceRNAs (13 lncRNAs, hsa-miR-365a-3p and BMP2; 13 lncRNAs, hsa-let-7e-5p and BMP2; 11 lncRNAs, hsa-miR-98- 5p and BMP2). Then, 4 lncRNAs (TUG1, LINC00689, NNT-AS1 and ZEB1-AS1), 2 lncRNAs (SNHG16 and AL590666.2), and 4 lncRNAs (PXN-AS1, ZEB1-AS1, TUG1 And NNT-AS1) were screened out separately through the analysis of next three steps. Supplementary Figures S10A–J showed the results of the correlation and difference analyses for these 10 lncRNAs. Supplementary Figure S10K showed the Kaplan-Meier survival curves of these seven lncRNAs.

We obtained 2 common miRNAs from 59 PR-DE-miRNAs of TCGA set, 92 DE-miRNAs of GSE138764 set, and 52 miRNAs of starBase (Supplementary Figure S11A). Supplementary Figures S11B–E showed the results of difference and survival analyses corresponding to these two miRNAs. 11/1 common circRNAs were obtained from 269 DE-circRNAs of the GSE165926 set and 6618/3647 circRNAs upstream of hsa-miR-129-5p/hsa-miR-381-3p predicted by starBase, respectively (Supplementary Figures 11F,G). Supplementary Figure S11H visualizes the differential expression results of these 12 circRNAs. The mechanisms by which miRNAs regulate target gene expression suggest that there should be negative correlations between miRNAs and BMP2, and between miRNAs and circRNAs. Only hsa_circ_0004662 and hsa_circ_0007548, which were up-regulated in gliomas, satisfied this regulation. Finally, the regulatory network composed of hsa_circ_0004662/hsa_circ_0007548, hsa-miR-129-5p and BMP2 was shown in Supplementary Figure S11I.

### Clinical Application of Prognostic Model

**FIGURE 11:**
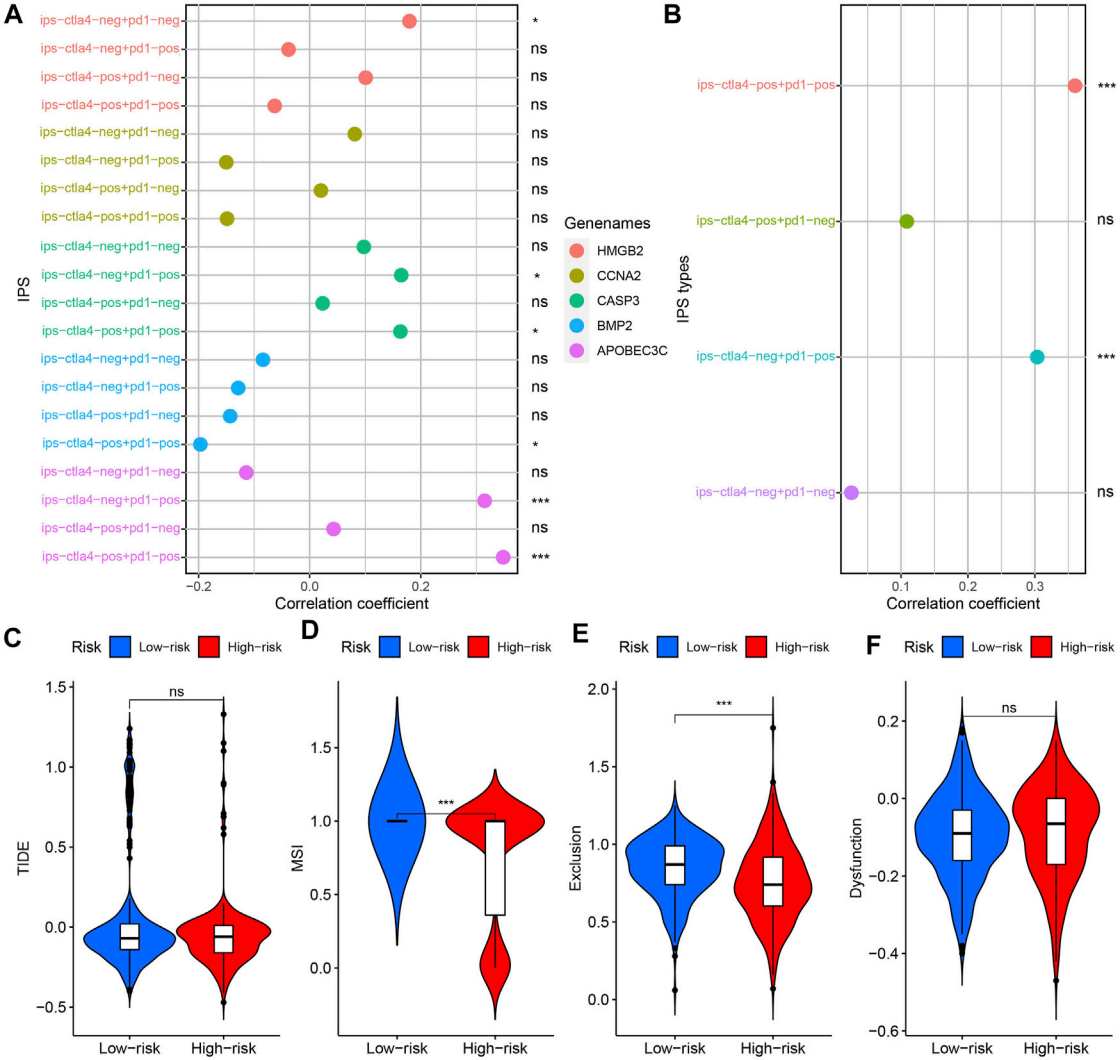
Model’s guiding value in ICIs therapy. **(A)** Correlation analysis results between 4 kinds of IPSs and 5 PR-DE-IRGs. **(B)** Correlation analysis results between 4 kinds of IPSs and risk score. **(C**–**F)** Difference in TIDE, MSI, T cell dysfunction and exclusion scores between high-risk and low-risk groups. ns: meaningless; *p < 0.05; **p < 0.01; ***p < 0.001.

IPS is used to assess which patients are more inclined to respond to ICIs therapy. A higher IPS evaluation value implies a better therapeutic effect of ICIs (Liu et al., 2020b). IPS showed broad correlation with risk score, CASP3, APOBEC3C, HMGB2, and BMP2 expression (Figures 11A,B). Such results suggested that BMP2, CASP3, APOBEC3C, HMGB2 and risk score can be used to predict the efficacy of ICIs. TIDE score was negatively correlated with the therapeutic effect of ICIs (Liu et al., 2020b). Figures 11C,F showed that there was no significant difference in TIDE score and T cell dysfunction score between the high-risk and low-risk groups. But, the T cell exclusion and MSI scores in the low-risk group were higher (Figures 11D,E).

**FIGURE 12:**
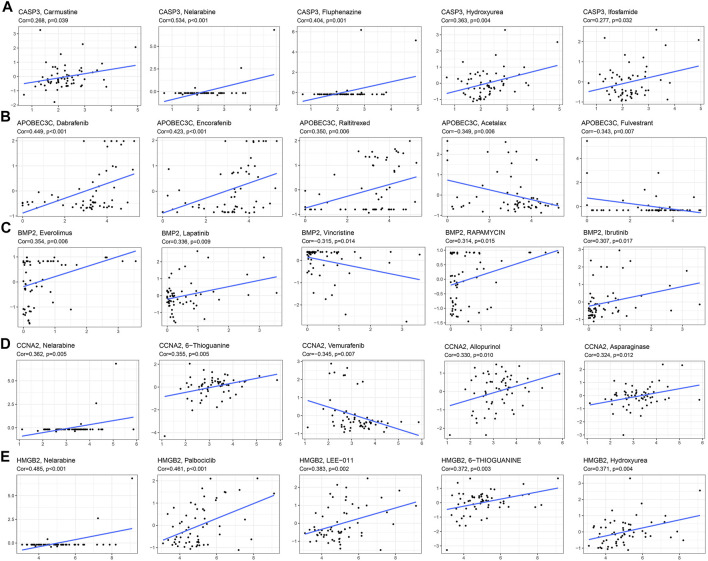
Correlation analysis between 5 PR-DE-IRGs and the 5 drugs approved by the food and drug administration (FDA). **(A)** CASP3. **(B)** APOBEC3C. **(C)** BMP2. **(D)** CCNA2. **(E)** HMGB2.

The correlation analysis results of the 5 drugs most closely related to 5 PR-DE-IRGs were visualized, respectively (Figures 12A–E). These results implied the guiding value of the five PR-DE-IRGs in these drugs’ clinical application. Unexpectedly, the expression of CASP3 was positively correlated with the sensitivity of carmustine, a drug recommended by the latest National Comprehensive Cancer Network (NCCN) for the treatment of glioma (Figure 12A).

**FIGURE 13:**
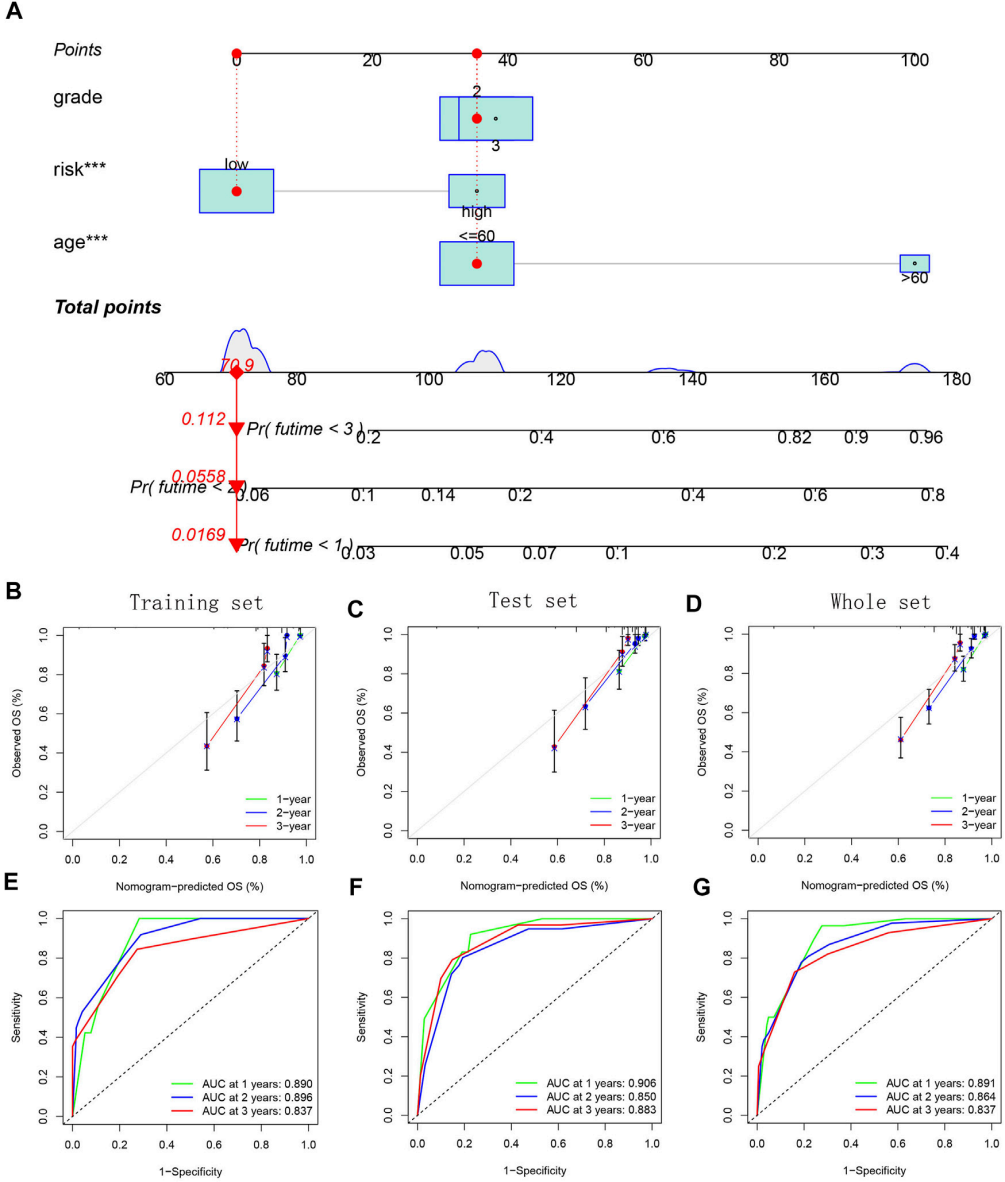
Establishment and verification of a combined nomogram. **(A)** Nomogram based on age, grade, and risk group. **(B**–**D)** Internal calibration curves for verifying model’s prediction accuracy. **(E**–**G)** ROC curves for evaluating model’s prediction performance.

Finally, we built a nomogram based on age, grade and risk score to predict the glioma patients’ survival probability (Figure 13A). The internal calibration curves showed that the predicted results were basically consistent with the actual results (Figures 13B–D). The ROC curves further confirmed the excellent predictive performance of the nomogram (AUC >0.8) (Figures 13E–G).

### Immune Checkpoint Inhibitors/N6-Methyladenine/Multidrug-Resistance Related Gene Expression Analysis

**FIGURE 14:**
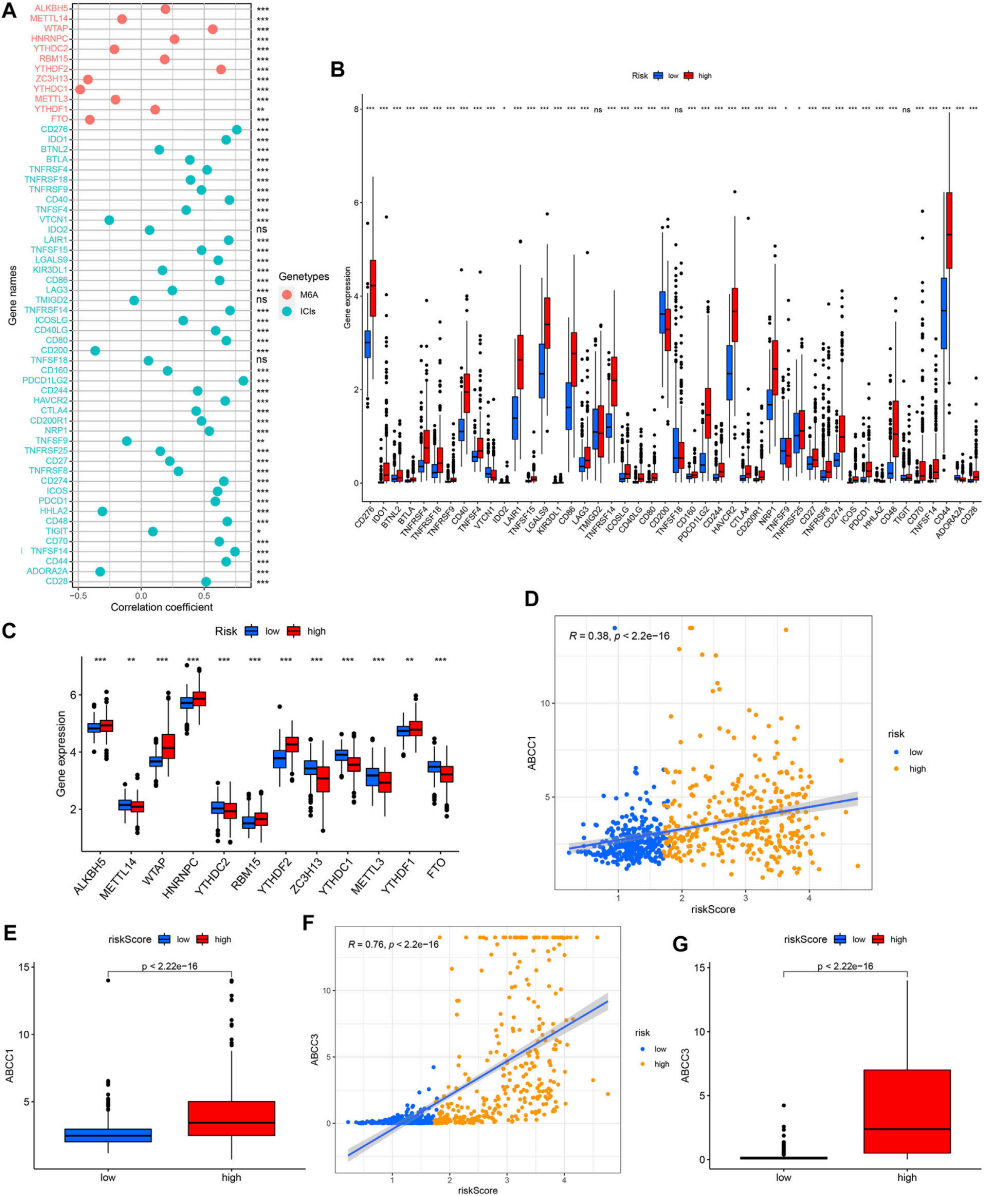
Correlation analysis between risk score and ICIs-related genes/m6a-related genes/multidrug resistance related genes expression, and comparison of them between different risk groups. **(A**,**B)** ICIs-related genes. **(A**,**C)** M6a-related genes. **(D**,**E)** ABCC1. **(F**,**G)** ABCC3.

Except for VTCN1, CD200, TNFSF9, HHLA2, and ADORA2A, which were negatively correlated with the risk score, the expressions of the other ICIs-related genes were all positively correlated with the risk score (Figure 14A). We also observed that half of the m6A-related genes’ expression were positively correlated with risk score, while the other half were negatively associated with the risk score (Figure 14A). The results of the above ICIs-related and m6A-related genes were verified in the further difference analysis (Figures 14B,C). We observed a positive correlation between ABCC1/ABCC3 expression and risk score as well as their higher expression in the high-risk group, further supporting the potential value of the model in drug resistance prediction (Figures 14D–G).

### Clinical Stratification Analysis Based on Prognostic Model

**FIGURE 15:**
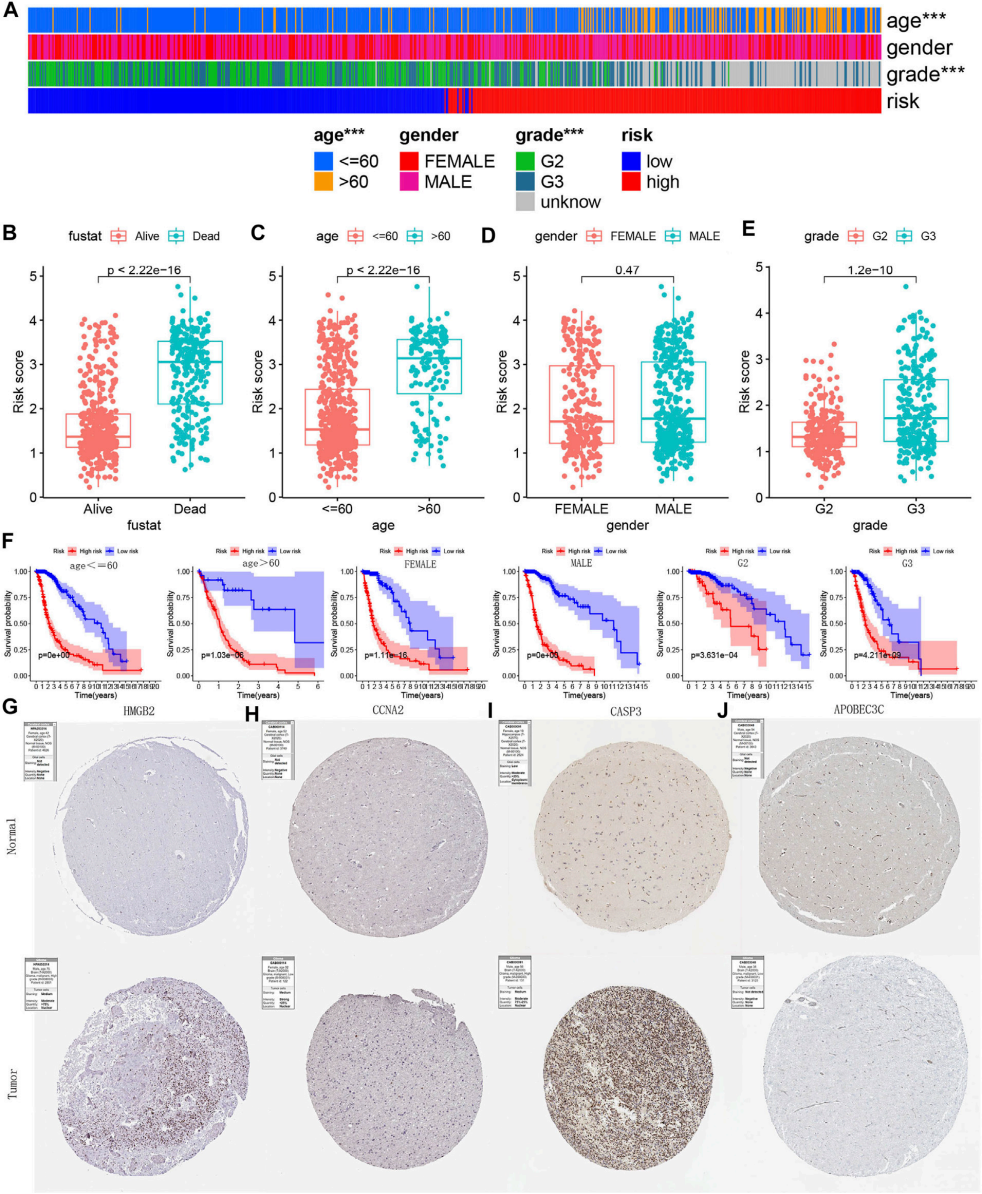
Clinical stratification analysis and IHC staining images verification. **(A)** Heatmap reflecting the clinical characteristics of each sample in TCGA. no asterisk: no significance; ***p < 0.001. **(B**–**E)** Risk score difference of between different clinical subgroups. **(F)** Kaplan-Meier survival curves reflecting the model’s ability to discriminate prognosis in each clinical subgroup. **(G**–**J)** IHC staining images reflecting the protein expression levels of HMGB2, CCNA2, CASP3, and APOBEC3C in glioma and normal brain tissue.

The heat map showed the distribution of clinical characteristics across all TCGA samples from different risk groups (Figure 15A). Subsequently, we further explored the relationship between survival status (Figure 15B)/age (Figure 15C)/gender (Figure 15D)/grade (Figure 15E) and risk score. The risk score of patients in the deceased group, age >60 group and G3 group were higher (Figures 15B,C,E). Figure 15F showed that the model still maintained the excellent performance of discriminating prognosis in all clinical characteristics subgroups. Figures 15G–J showed the IHC staining images reflecting the protein expression levels of the four PR-DE-IRGs (HMGB2, CCNA2, CASP3, and APOBEC3C) in the model, respectively. We only observed higher expression of three genes (HMGB2, CCNA2, and CASP3) in the glioma tissues (Figures 15G–I), consistent with the conclusions from the previous analysis based on the TCGA cohort. The above results further verified the model’s stability.

### Immunohistochemical Staining Images Verification

Figures 15G–J showed the IHC staining images reflecting the protein expression levels of the four PR-DE-IRGs (HMGB2, CCNA2, CASP3 and APOBEC3C) in the model, respectively. We only observed higher expression of three genes (HMGB2, CCNA2 and CASP3) in the glioma tissues (Figures 15G–I), consistent with the conclusions from the previous analysis based on the TCGA cohort. The above results further verified the model’s stability.

## Discussion

This study extracted multiple datasets from TCGA, GEO, and CGGA databases for analysis. WGCNA identified 41 DE-IRGs highly associated with gliomas. The 15 PR-DE-IRGs screened out by prognostic analysis were used in lasso regression analysis. Finally, five PR-DE-IRGs highly associated with glioma were used to construct a prognostic model. Kaplan-Meier analysis, multivariate Cox regression and ROC curves validated the excellent performance of risk score as an independent predictor in predicting prognosis. In addition to external cohorts, the data from clinical subgroups also supported this conclusion. The IHC staining images of HMGB2, CCNA2, and CASP3 demonstrated their differences in protein expression between glioma and normal brain tissues, further supporting the stability of the model. Through correlation analysis, we determined the excellent value of the model in guiding immunotherapy and chemotherapy. In addition, integrating the analysis of Muti-Omics data, we also found many results closely related to the progression and prognosis of glioma in terms of cell stemness, ceRNA regulatory axis, mutation and tumor immunosuppressive microenvironment.

Combined with previous studies, 5 genes are closely related to numerous cancers and immunity (Kwon et al., 2010; Galluzzi et al., 2016; Gan et al., 2018; Bernard et al., 2019; Constantin et al., 2021). Cyclin A2 (CCNA2) is a cyclin gene that acts as a biomarker for various tumors (Hydbring et al., 2016; Gan et al., 2018; Guo et al., 2020; Wang et al., 2020). Cancer cells, such as colorectal cancer, esophageal carcinoma, and ovarian cancer, can be inhibited from growing and progressing through the cell cycle by the silent CCNA2 (Gan et al., 2018; Guo et al., 2020; Wang et al., 2020). In particular, the study of Xi et al. showed that the invasion and metastasis of gliomas could be inhibited by reducing the expression of CCNA2 protein (Xi et al., 2019). HMGB2 belongs to the high mobility group box (HMGB) protein family, which can participate in the innate immune response of mammalian nucleic acid mediators (Rao et al., 2013; Morinaga et al., 2021). In addition, HMGB2 has been proved to be an independent influencing factor of the patient’s prognosis by its protein or mRNA level in hepatocellular carcinoma, epithelial ovarian cancer and glioblastoma multiforme (Ouellet et al., 2006; Kwon et al., 2010; Wu et al., 2013). Caspase 3 (CASP3) belongs to the caspase protease family, whose activation can cause the cleavage of many important functional proteins in cells, thereby achieving the purpose of apoptosis (Eckhart et al., 2005; Galluzzi et al., 2016). Furthermore, Bernard et al.’s study showed that the inhibition of CASP3 can reduce the development of spontaneous tumors and make cells sensitive to chemotherapeutics (Bernard et al., 2019). Apolipoprotein B mRNA-editing catalytic polypeptide-like 3C (APOBEC3C), a member of the APOBEC3 subfamily, is implicated in HIV-1 restriction, with an unclear relationship to cancer (Refsland et al., 2010; Constantin et al., 2021). As a member of the transforming growth factor-β super-family, enhanced expression of bone morphogenetic protein 2 (BMP2) can check the growth of colorectal cancer cells, enhance apoptosis, and reduce tumor development in the body (Vishnubalaji et al., 2016; Zhou et al., 2016). The conclusions of the above studies all support the accuracy of our analysis results.

A growing body of research has revealed that ceRNA regulatory networks are closely associated with the pathogenesis of glioma. The members that emerged in our ceRNA network were all closely related to tumors, and a large proportion was closely related to gliomas. The hsa-let-7e-5p was found to be a tumor suppressor to inhibit the progression of head and neck squamous cell carcinoma by targeting CCR7 expression (Wang et al., 2019) and a potential prognosis marker for rectal carcinoma with liver metastases (Chen et al., 2018). Many previous studies have also demonstrated that hsa-miR-365 functions as a tumor-suppressor in various cancers (Nie et al., 2012; Wang Y. et al., 2017). Li et al. revealed that ZEB1-AS1 interacts with hsa-miR-365a-3p and inhibits hsa-miR-365a-3p function (Li et al., 2019). Interestingly, both hsa-let-7e-5p and hsa-miR-98-5p belong to a tumor suppressor family of miRNA, the let-7 family, which is down-regulated in many tumor types and is closely related to tumorigenesis (Lu et al., 2005; Croce, 2009; Blandino et al., 2014). The expression level of let-7 is positively correlated with the malignant grade of gliomas, indicating that let-7 is likely to be a crucial inhibitory factor in the progression of gliomas (Lee et al., 2011). The abnormal expression of hsa-miR-365a-3p has been reported in many tumors, and it is also a tumor suppressor gene in gastric cancer (Guo et al., 2013; Hamada et al., 2014). Hsa-miR-129-5p, a tumor suppressor, is down-regulated in gliomas and inhibits glioma proliferation, migration and development by targeting TGIF2, WNT5A, DNMT3A, HOXC10, FNDC3B (Xu et al., 2017; Gu et al., 2018; Zeng et al., 2018; Liu et al., 2020a). The relationship between BMP2 and hsa-miR-129-5p has been reported in many diseases, such as hepatocellular carcinoma (Liu et al., 2021) and intervertebral disc degeneration (Yang and Sun, 2019). Liu et al. proved that BMP2 is the target gene of hsa-miR-129-5p and is post-transcriptional regulated by miR-129-5p (Liu et al., 2021). All these researches support our analytics results.

Previous studies have shown that lncRNAs are involved in glioma progression and prognosis (Kiang et al., 2015; Peng et al., 2018; Xi et al., 2018). The aberrant expression of taurine up-regulated gene 1 (TUG1) is associated with cell proliferation, migration, cell cycle changes, apoptosis and drug resistance in numerous tumors (Khalil et al., 2009). In glioma, TUG1 functions as a tumor suppressor by promoting cell apoptosis via activating caspase-3 and caspase-9 mediated intrinsic pathways and inhibiting Bcl-2 mediated anti-apoptotic pathways (Li et al., 2016). Additionally, small nucleolar RNA host gene 16 (SNHG16) suppressed the expression of p21, caspase-3 and caspase-9, while promoting cyclin-D1 and cyclin-B1 expression to inhibit apoptosis in glioma cells (Zhou et al., 2020). CircRNAs can recruit other RNA species, and affect the transcriptional silencing, translation and/or decay of specific mRNAs through the binding of miRNAs (Patop et al., 2019). Han et al. observed a high expression of hsa_circ_0004662 in hepatocellular carcinoma, suggesting its important role in the occurrence and development of hepatocellular carcinoma (Han et al., 2022). The crosstalk based on shared MREs in RNA transcript forms a complex network. The expression of lncRNAs and circRNAs alters the levels of miRNAs, which in turn affects the expression of their target mRNAs (Yang et al., 2014; Panda, 2018). Theoretically, the expression level of lncRNA/circRNA is negatively correlated with that of miRNA, and our results are highly consistent with this (Gawronski et al., 2018; Goodall and Wickramasinghe, 2021). Our results suggest that the down-regulation of miRNAs as suppressors in ceRNAs leads to up-regulation of the protective factor BMP2 and the corresponding lncRNAs and circRNAs. Compared with studying the isolated ceRNA axis, the analysis of a more extensive interconnected ceRNA network may be closer to physiological conditions and is conducive to more profound insight into ceRNA-mediated gene regulation. In our study, the mRNAs, miRNAs, and lncRNAs in the network showed a significant correlation with the prognosis of glioma, suggesting that miRNAs, circRNAs and lncRNAs may affect mRNAs expression in a combined way involving multiple pathways, thus affecting the progression and prognosis of the tumor.

Except for activated dendritic cells (aDCs), mast cells, and NK cells, the remaining 13 immune cells were more abundant in the high-risk group, which further supported the observation in the GSEA enrichment analysis that the high-risk group covered more immune-related biological functions and pathways. Neutrophils and macrophages play a vital role in regulating the immune response to inflammation in cancer (Galdiero et al., 2013). The presence of neutrophils at melanoma ulcer sites is strongly associated with the cell proliferation at these sites, which is associated with poor prognosis (Antonio et al., 2015). When macrophages are exposed to T helper 2 (Th2) cytokines (such as IL-4 and IL-13), they polarize into M2 macrophages and promote tumor growth, while tumor-associated macrophages (TAMs) are mainly characterized by M2 macrophages (Galdiero et al., 2013).

Hambardzumyan et al. found that TAMs interact with tumor cells, providing a conducive microenvironment that allows the tumor to escape from immune detection, thereby promoting the proliferation and metastasis of gliomas (Hambardzumyan et al., 2016). Plasmacytoid dendritic cells (pDCs) are responsible for creating an immunosuppressive microenvironment in various tumors (Vermi et al., 2011). Peritoneal pDCs infiltration may represent an immune pathogenic pathogen microenvironment and can be used to predict poor prognosis in patients undergoing therapeutic profiling for intrahepatic cholangiocarcinoma (Hu et al., 2020). The above analyses suggest that the poor prognosis of high-risk groups may be closely related to the tumor immunosuppressive environment generated by these immune cells. According to previous reports, IDH1 mutation is common in many malignant tumors, such as glioma, acute myeloid leukemia, thyroid cancer and chondroma (Murugan et al., 2010; Amary et al., 2011; Losman et al., 2013; Bai et al., 2016). Studies have shown that mutation in the IDH1 gene, especially the R132H mutation, can promote NK cell recruitment through CX3CL1/CX3CR1 chemotherapy and are associated with a better prognosis in gliomas (Ren et al., 2019). This finding suggests that NK cells can be enriched in the IDH1 mutant group, suggesting a good prognosis for glioma, which coincides with our results. Furthermore, this may explain why NK cells were negatively correlated with risk score in our study. BMP2 gene was expressed more in the IDH1 mutant group. Blood circulating NK cells express type I and type II BMP receptors, BMP-2 and BMP-6 ligands, which mediate the signaling of the BMP family members (Robson et al., 2014). The results of the study by Robson et al. showed that the inhibition of BMP signal could effectively inhibit the effect or function of NK cells, providing a new idea for immunotherapy to kill tumor cells (Robson et al., 2014). Through the above studies, we speculate that the better prognosis of patients with IDH1 mutation is due to the fact that BMP signaling pathway can stimulate the effect or function of NK cells.

As the results show, our model has excellent capabilities in predicting the prognosis of patients with glioma and guiding clinical treatment. However, the research is not perfect and still has numerous limitations. For example, the difference in protein expression of APOBEC3C is still not supported by IHC images obtained based on experiments. Moreover, this study did not combine basic experiments to verify the results of the study. Still, it only used the results of others to explain and further speculate on our results rationally. Even if limited by these shortcomings, the rich mechanism discussion results may provide new directions for follow-up research.

## Data Availability

The original contributions presented in the study are included in the article/Supplementary Material, further inquiries can be directed to the corresponding author.

## Supplementary Material

Supplementary Figure S1 Difference analysis results of IRGs from the TCGA cohort. **(A)** Heatmap showing the expression levels of the top 100 DE-IRGs in glioma and normal tissues. **(B)** Volcano plot showing the expression status of all IRGs. The red/black/green dots represent up-regulated genes/genes with no significant difference/down-regulated genes, respectively.

Supplementary Figure S2 Difference analysis results of IRGs from the GSE108474 cohort. **(A)** Heatmap showing the expression levels of the top 100 DE-IRGs in glioma and normal tissues. **(B)** Volcano plot showing the expression status of all IRGs. The red/ black/green dots represent up-regulated genes/genes with no significant difference/down-regulated genes, respectively.

Supplementary Figure S3 Difference analysis results of IRGs from the GSE4290 cohort. **(A)** Heatmap showing the expression levels of the top 100 DE-IRGs in glioma and normal tissues. **(B)** Volcano plot showing the expression status of all IRGs. The red/black/green dots represent up-regulated genes/genes with no significant difference/down-regulated genes, respectively.

Supplementary Figure S4 PR-DE-IRGs shared by the TCGA, CGGA, GSE4412, and GSE43378 cohorts. **(A)** Venn diagram showing the overlapping process of PR-DE-IRGs from 4 cohorts. **(B**–**D)** Forest plot showing the univariate Cox regression analysis results of 15 PR-DE-IRGs from CGGA, GSE4412, and GSE43378 cohorts, respectively.

Supplementary Figure S5 Survival curves of 15 PR-DE-IRGs from the CGGA cohort.

Supplementary Figure S6 Survival curves of 15 PR-DE-IRGs from the GSE4412 cohort.

Supplementary Figure S7 Survival curves of 15 PR-DE-IRGs from the GSE43378 cohort.

Supplementary Figure S8 The process of determining 5 PR-DE-IRGs and corresponding coefficients based on the optimal penalty parameter (*λ*) *via* Lasso regression. **(A)** The penalty coefficient was utilized to minimize the mean square error of the model. **(B)** LASSO regression model profile for the 5 selected PR-DE-IRGs.

Supplementary Figure S9 PCA and tSNE dot plots based on the training, testing, whole, CGGA, GSE4412, and GSE43378 sets.

Supplementary Figure S10 Corresponding analysis results of 10 screened lncRNAs. **(A**–**D)** Correlation and difference analysis results of TUG1, LINC00689, NNT-AS1, and ZEB1-AS1 upstream of hsa-miR-365a-3p. **(E**,**F)** Correlation and difference analysis results of SNHG16 and AL590666.2 upstream of hsa-let-7e-5p. **(G**–**J)** Correlation and difference analysis results of PXN-AS1, ZEB1-AS1, TUG1, and NNT-AS1 upstream of hsa-miR-98-5p. **(K)** Kaplan-Meier survival curves of TUG1, LINC00689, NNT-AS1, ZEB1-AS1, SNHG16, AL590666.2, and PXN-AS1.

Supplementary Figure S11 Construction of the regulatory network consisting of circRNAs, miRNAs, and BMP2. **(A)** Obtaining common miRNAs by Venn diagram. **(B**,**C)** Differential expression results of hsa-miR-129-5p and hsa-miR-381-3p based on GSE138764 set and TCGA set, respectively. **(D**,**E)** Kaplan-Meier survival curves for hsa-miR-129-5p and hsa-miR-381-3p based on the TCGA set, respectively. **(F**,**G)** Obtaining common circRNAs from 269 DE-circRNAs of the GSE165926 set and 6618/3647 circRNAs upstream of hsa-miR-129-5p/hsa-miR-381-3p predicted by starBase, respectively. **(H)** Differential expression results of 12 common circRNAs based on GSE165926 set. **(I)** The regulatory network consisting of hsa_circ_0004662/hsa_circ_0007548, hsa-miR-129-5p and BMP2. **p* < 0.05; ***p* < 0.01; ****p* < 0.001.
