# Supplementary figures and images for: An Integrated Immune-Related Bioinformatics Analysis in Glioma: Prognostic Signature’s Identification and Multi-Omics Mechanisms’ Exploration
Source: Front Genet. 2022 May 3;13:889629. doi: 10.3389/fgene.2022.889629 (PMC9114310; doi:10.3389/fgene.2022.889629)

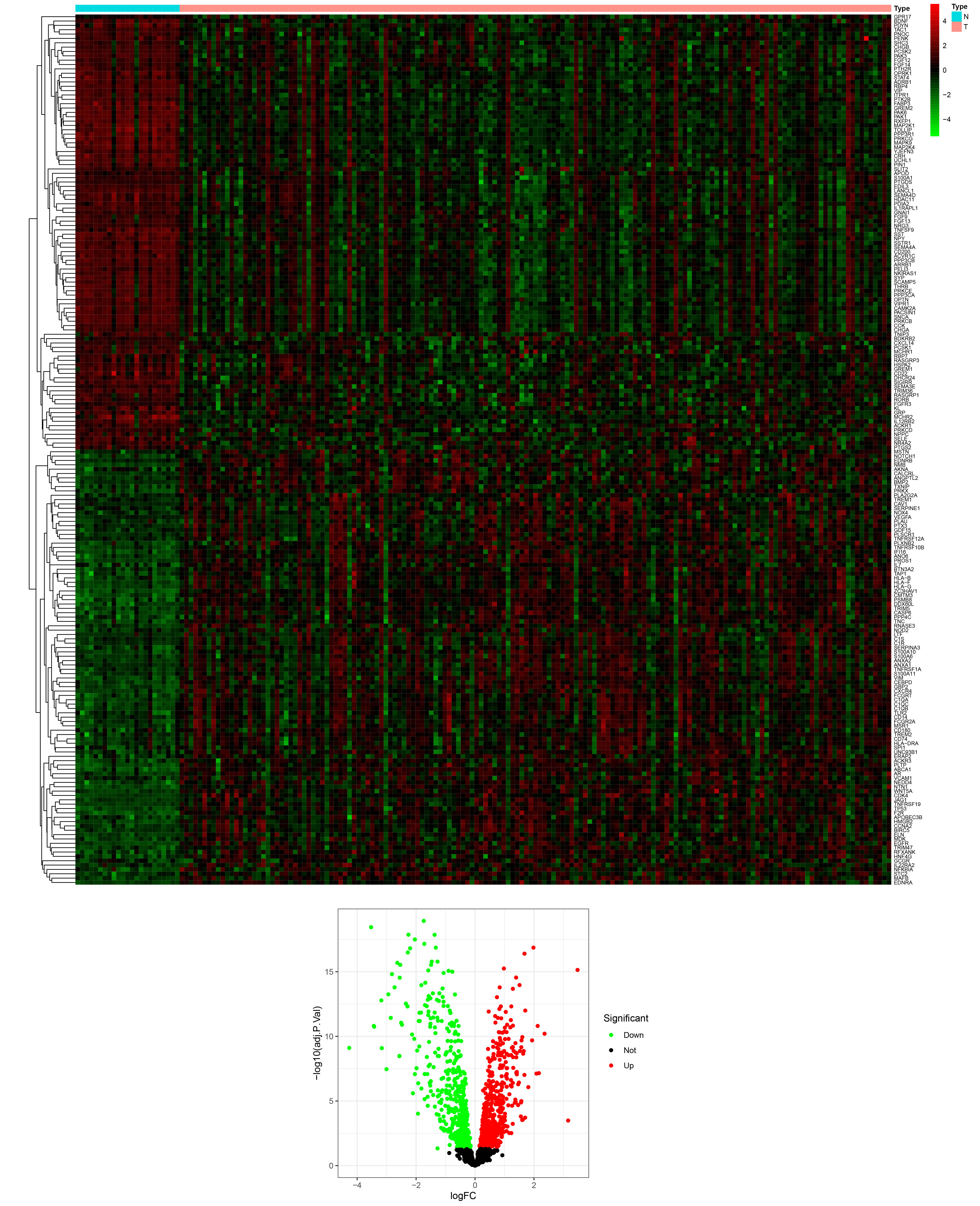

Supplement: Supplementary file 1 [file Image3.JPEG]

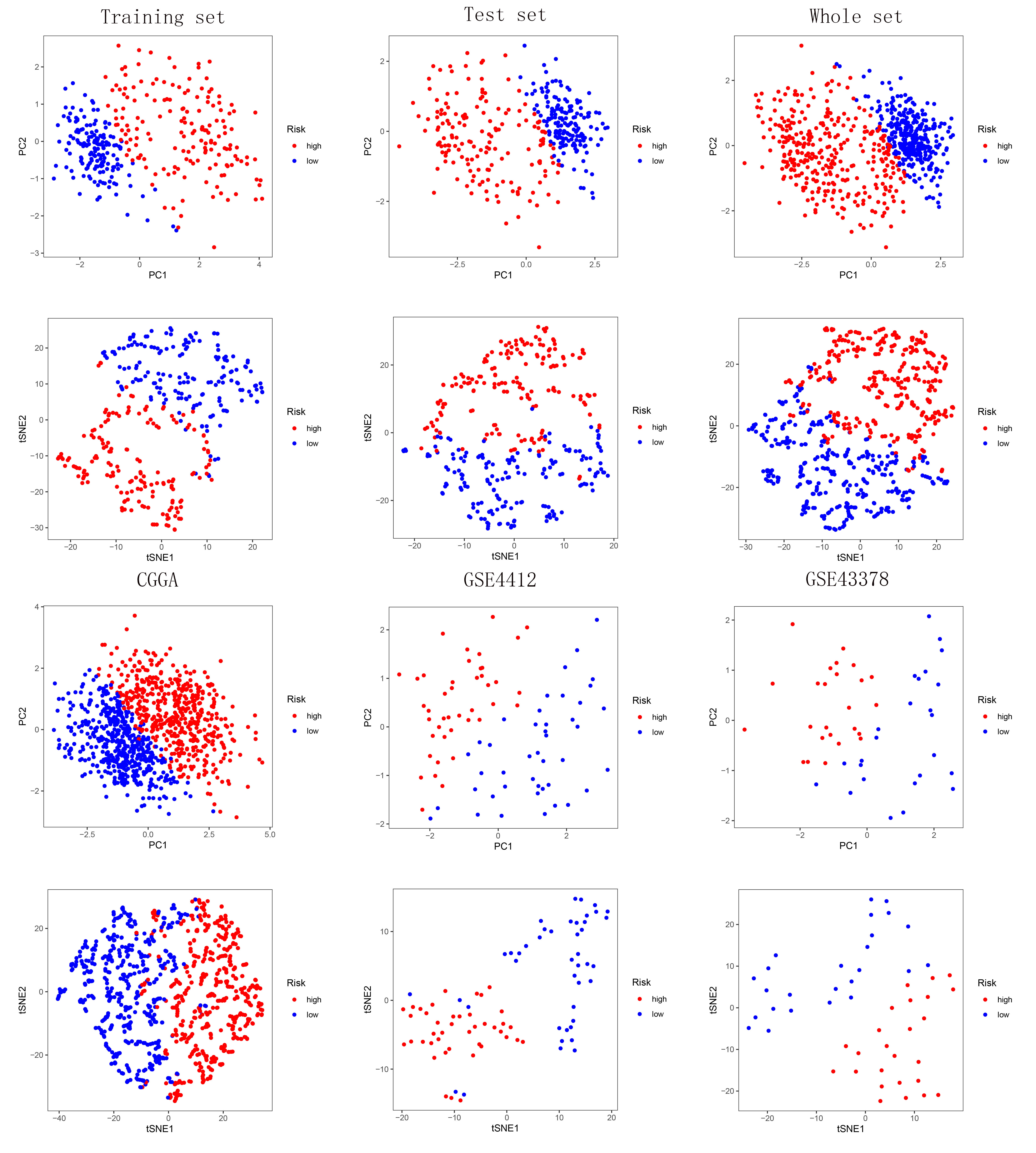

Supplement: Supplementary file 3 [file Image9.JPEG]

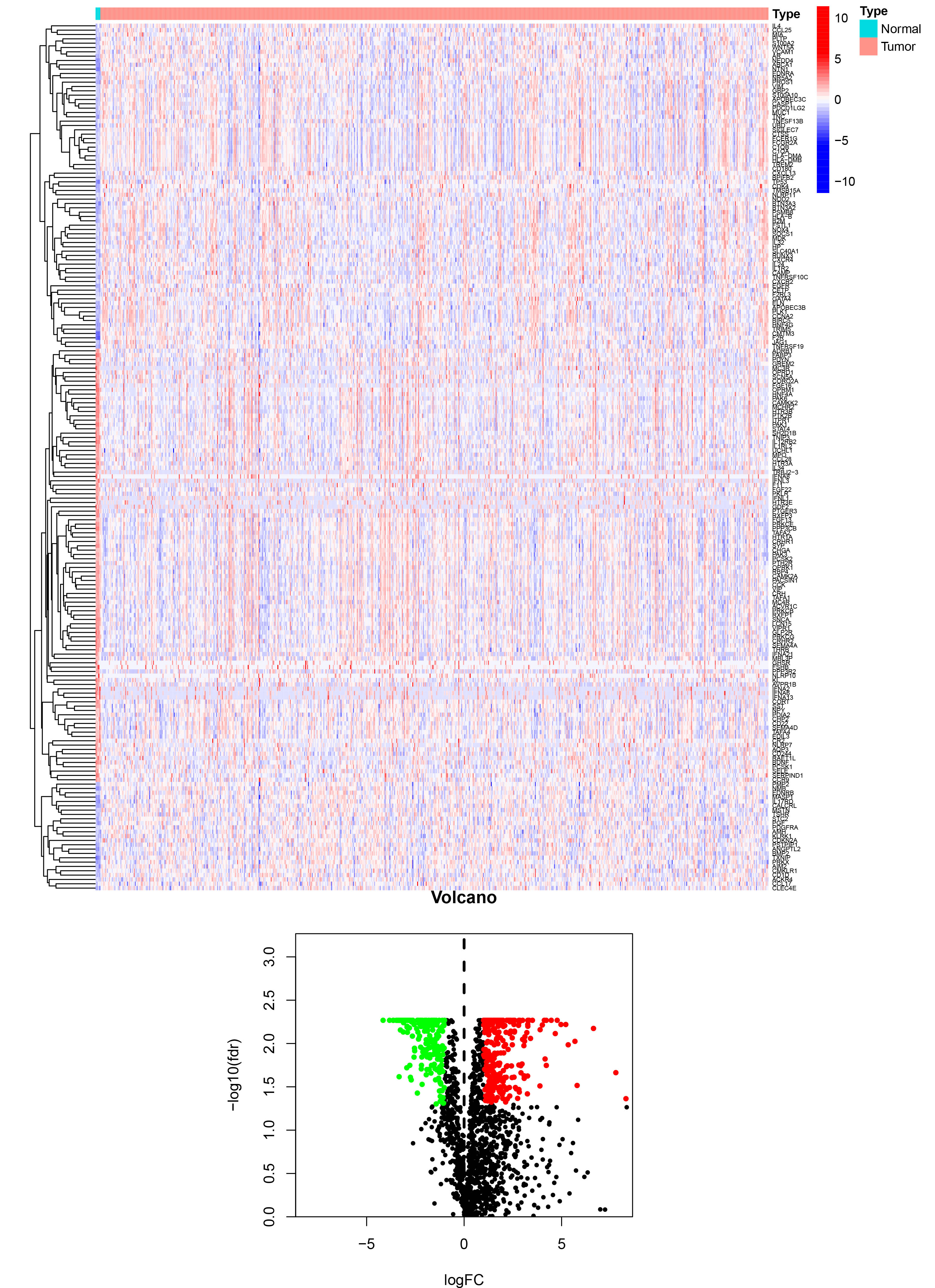

Supplement: Supplementary file 4 [file Image1.JPEG]

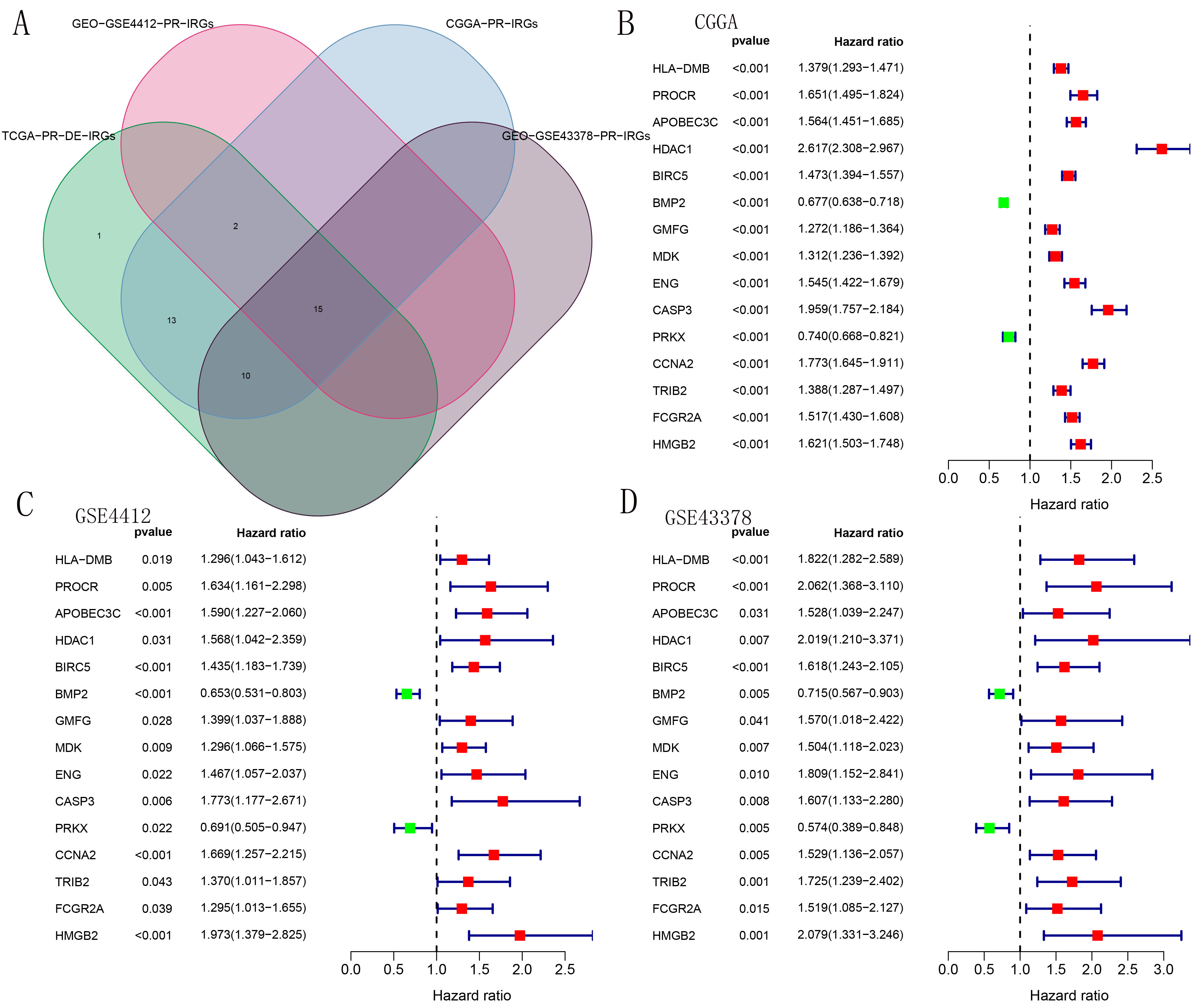

Supplement: Supplementary file 5 [file Image4.JPEG]

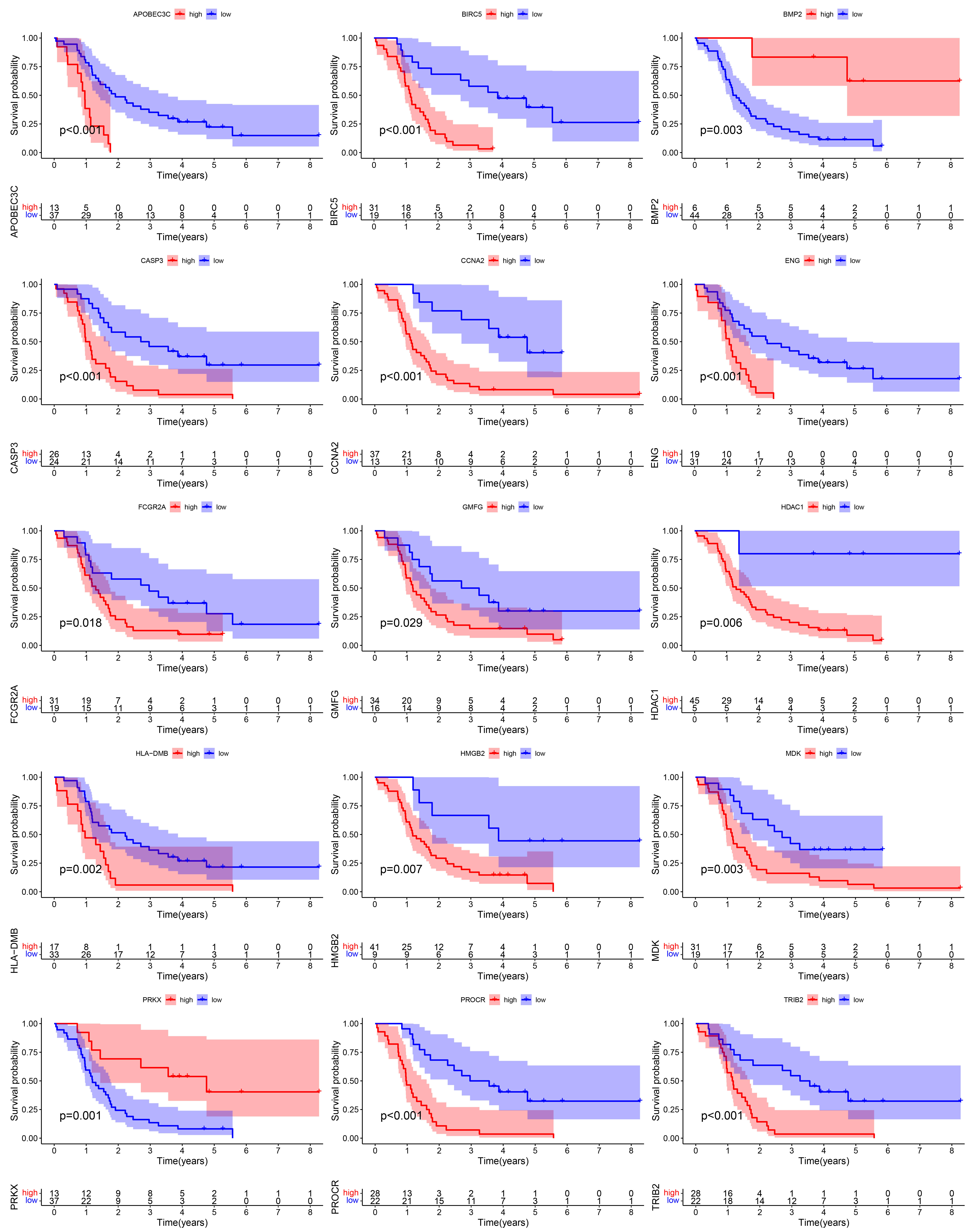

Supplement: Supplementary file 6 [file Image7.JPEG]

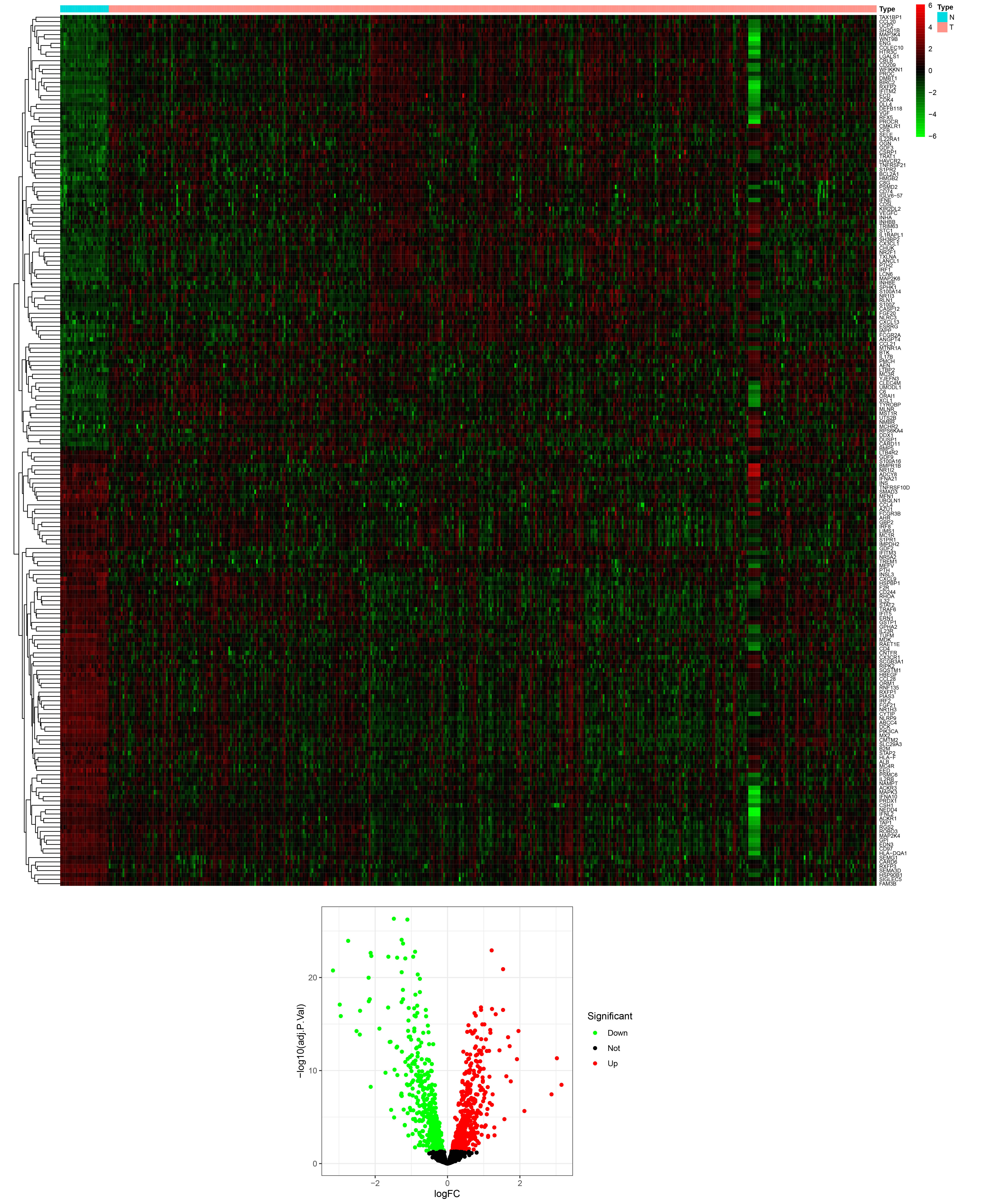

Supplement: Supplementary file 7 [file Image2.JPEG]

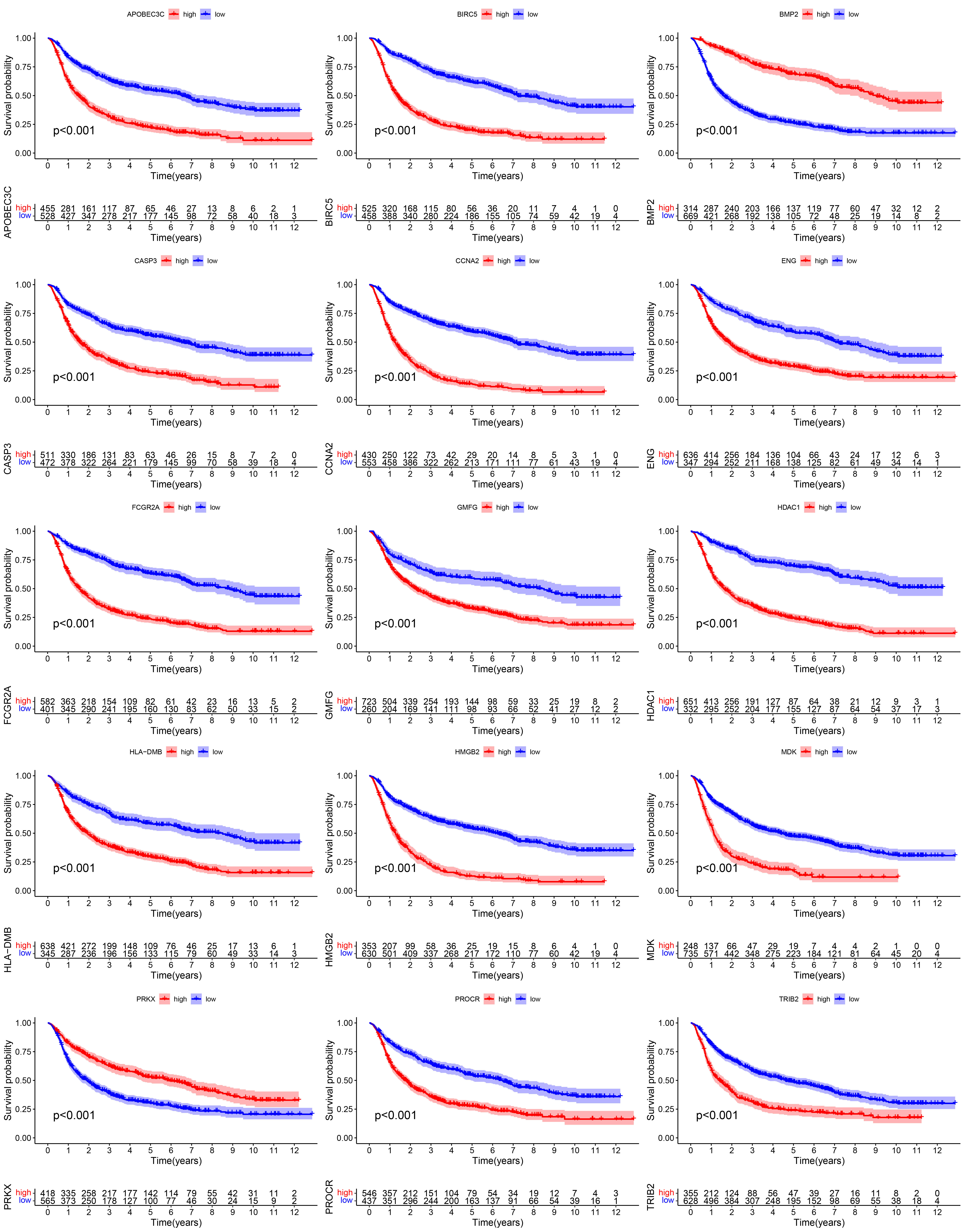

Supplement: Supplementary file 8 [file Image5.JPEG]

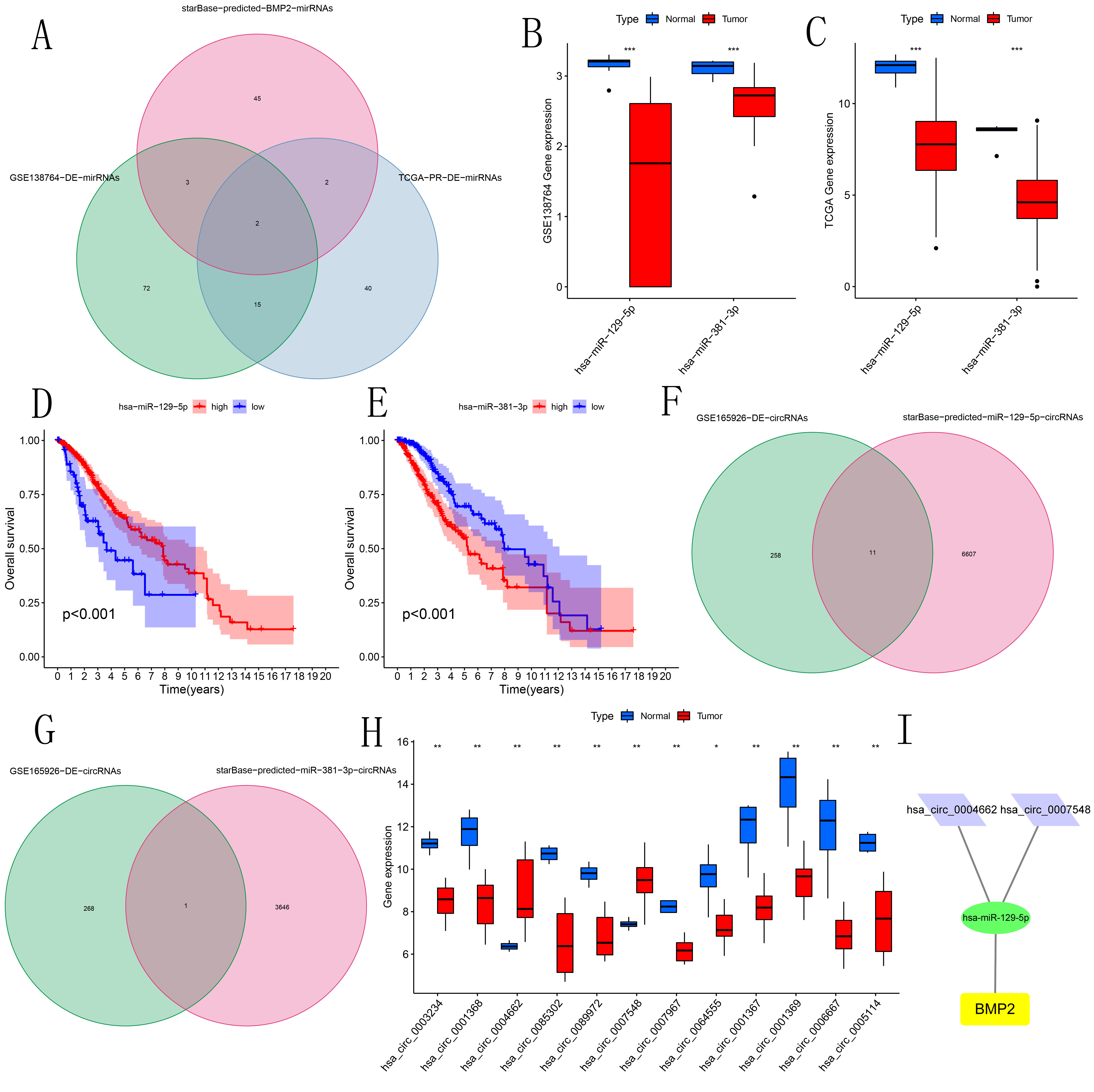

Supplement: Supplementary file 10 [file Image11.JPEG]

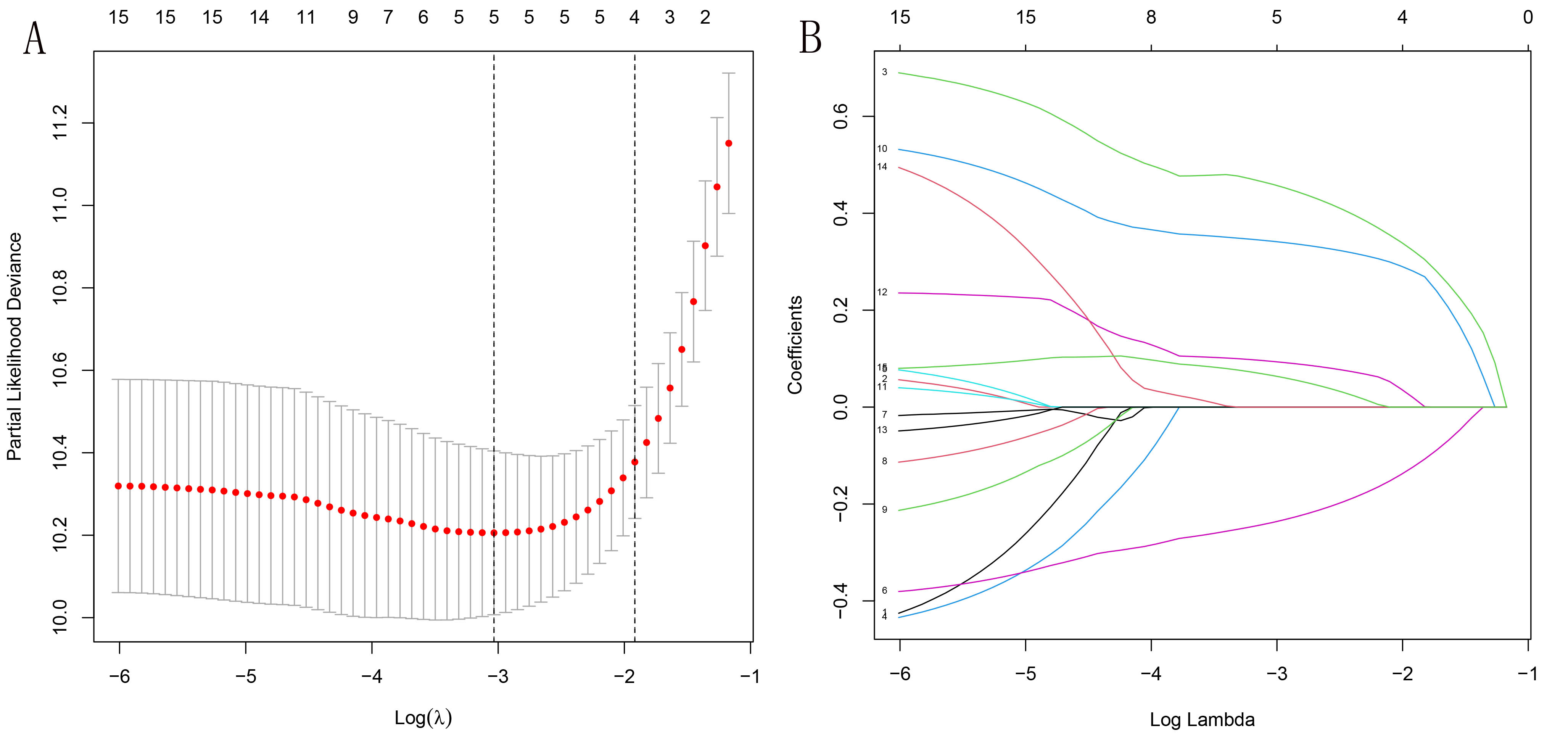

Supplement: Supplementary file 14 [file Image8.JPEG]

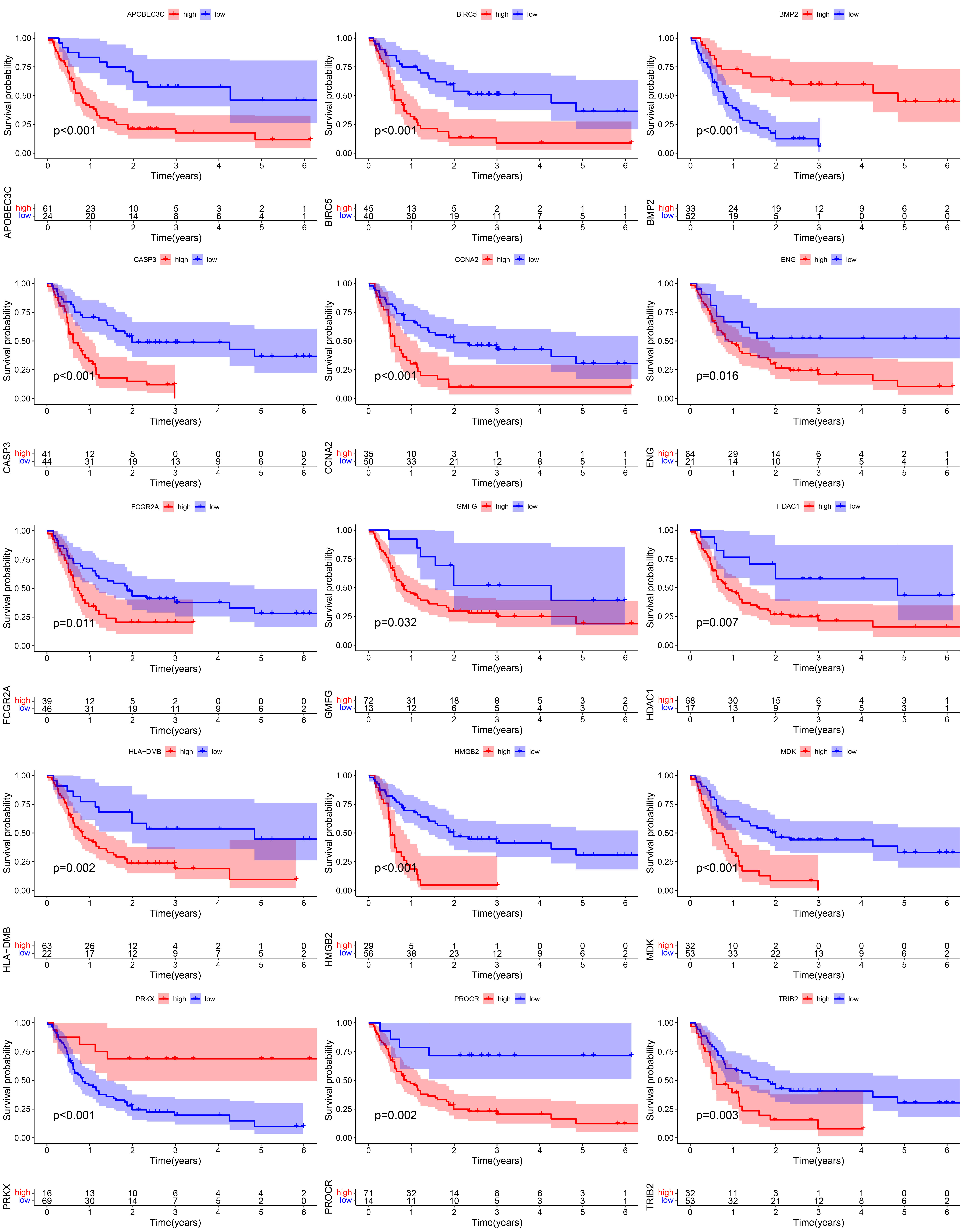

Supplement: Supplementary file 15 [file Image6.JPEG]
