# Supplementary material for: An Integrated Immune-Related Bioinformatics Analysis in Glioma: Prognostic Signature’s Identification and Multi-Omics Mechanisms’ Exploration
Source: Front Genet. 2022 May 3;13:889629. doi: 10.3389/fgene.2022.889629 (PMC9114310; doi:10.3389/fgene.2022.889629)
Supplement: Supplementary file 2 [file Table1.DOCX]

**Supplementary Table 1. Clinical characteristics of glioma samples in training, test, whole sets from TCGA**

|  | **Training set (n=334)** | **Test set (n=334)** | **Whole set (n=668)** |
| --- | --- | --- | --- |
| **Gender (%)** | | | |
| Male | 179(53.59%) | 206(61.68%) | 385(57.63%) |
| Female | 155(46.41%) | 128(38.32%) | 283(42.37%) |
| **Age (%)** | | | |
| ≤60 | 275(82.34%) | 254(76.05%) | 529(79.19%) |
| ＞60 | 59(17.66%) | 80(23.95%) | 139(20.81%) |
| **Survival status** | | | |
| OS-days (median，range) | 548(0-6423) | 575(0-5546) | 565(0-6423) |
| OS-state（alive(%)/  dead(%)） | 207(62.0%)/127(38.0%) | 207(62.0%)/127(38.0%) | 414(62.0%)/254(38.0%) |
| **Grade(%)** | | | |
| 2 | 131(39.22%) | 116(34.73%) | 247(36.98%) |
| 3 | 130(38.92%) | 131(39.22%) | 261(39.07%) |
| unknown | 73(21.86%) | 87(26.05%) | 160(23.95%) |
