# Supplementary material for: An Integrated Immune-Related Bioinformatics Analysis in Glioma: Prognostic Signature’s Identification and Multi-Omics Mechanisms’ Exploration
Source: Front Genet. 2022 May 3;13:889629. doi: 10.3389/fgene.2022.889629 (PMC9114310; doi:10.3389/fgene.2022.889629)
Supplement: Supplementary file 11 [file Table2.DOCX]

**Supplementary Table 2. Clinical characteristics of glioma samples in 2 GEO cohorts and CGGA cohort**

|  | **GSE4412 cohort (n=85)** | **GSE43378 cohort (n=50)** | **CGGA cohort (n=983)** |
| --- | --- | --- | --- |
| **Gender (%)** | | | |
| Male | 32(37.6%) | 34(68.0%) | 405(41.2%) |
| Female | 53(62.4%) | 16(32.0%) | 578(58.8%) |
| **Age (%)** | | | |
| ≤60 | 70(82.4%) | 29(58.0%) | 869(88.4%) |
| ＞60 | 15(17.6%) | 21(42.0%) | 113(11.5%) |
| unknown | 0(0.0%) | 0(0.0%) | 1(0.1%) |
| **Survival status** | | | |
| OS-days (median，range) | 389(7-2516) | 545(20-3020) | 777(19-4697) |
| OS-state（alive(%)/  dead(%)） | 26(30.6%)/59(69.4%) | 8(16.0%)/42(84.0%) | 387(39.4%)/596(60.6%) |
| **Grade(%)** | | | |
| 2 | 0(0.0%) | 5(10.0%) | 280(28.5%) |
| 3 | 26(30.6%) | 13(26.0%) | 325(33.1%) |
| 4 | 59(69.4%) | 32(64.0%) | 374(38.0%) |
| unknown | 0(0.0%) | 0(0.0%) | 4(0.4%) |
