# Supplementary material for: An Integrated Immune-Related Bioinformatics Analysis in Glioma: Prognostic Signature’s Identification and Multi-Omics Mechanisms’ Exploration
Source: Front Genet. 2022 May 3;13:889629. doi: 10.3389/fgene.2022.889629 (PMC9114310; doi:10.3389/fgene.2022.889629)
Supplement: Supplementary file 12 [file Table3.DOCX]

**Supplementary Table 3. The PR-DE-IRGs used to construct the model and the corresponding coefficients**

| **Gene** | **Coef** |
| --- | --- |
| **APOBEC3C** | 0.459669 |
| **BMP2** | -0.23796 |
| **CASP3** | 0.342148 |
| **CCNA2** | 0.094172 |
| **HMGB2** | 0.065041 |
