# Supplementary material for: An Integrated Immune-Related Bioinformatics Analysis in Glioma: Prognostic Signature’s Identification and Multi-Omics Mechanisms’ Exploration
Source: Front Genet. 2022 May 3;13:889629. doi: 10.3389/fgene.2022.889629 (PMC9114310; doi:10.3389/fgene.2022.889629)
Supplement: Supplementary file 13 [file Table4.DOCX]

**Supplementary Table 4. The results of correlation analysis and difference analysis of all predicted miRNAs**

| **Gene** | **miRNA** | **cor** | **pvalue** | **logFC** | **diffPval** |
| --- | --- | --- | --- | --- | --- |
| **BMP2** | hsa-let-7e-5p | -0.49451 | 0 | -2.08108 | 0.000348 |
| **BMP2** | hsa-miR-142-5p | -0.4529 | 0 | -0.69429 | 0.119593 |
| **BMP2** | hsa-miR-98-5p | -0.4403 | 0 | -1.74861 | 0.000155 |
| **BMP2** | hsa-let-7i-5p | -0.43119 | 0 | -0.43721 | 0.057504 |
| **BMP2** | hsa-miR-365a-3p | -0.40073 | 0 | -1.97594 | 0.000202 |
| **BMP2** | hsa-miR-654-3p | -0.35537 | 3.85E-17 | -2.4399 | 0.000654 |
| **BMP2** | hsa-miR-370-3p | -0.34846 | 2.20E-16 | -3.59409 | 0.0002 |
| **BMP2** | hsa-let-7d-5p | -0.34742 | 2.81E-16 | -1.39127 | 0.000219 |
| **BMP2** | hsa-miR-369-3p | -0.3408 | 9.60E-16 | -3.49051 | 0.000185 |
| **BMP2** | hsa-miR-654-5p | -0.33283 | 4.79E-15 | -1.8956 | 0.000438 |
| **BMP2** | hsa-miR-541-3p | -0.33096 | 6.93E-15 | -3.15082 | 0.000209 |
| **BMP2** | hsa-miR-381-3p | -0.31452 | 2.15E-13 | -3.60906 | 0.000232 |
| **BMP2** | hsa-miR-505-3p | -0.31373 | 2.48E-13 | 0.14354 | 0.577791 |
| **BMP2** | hsa-miR-543 | -0.31267 | 2.27E-13 | -3.03035 | 0.000191 |
| **BMP2** | hsa-miR-656-3p | -0.30433 | 1.03E-12 | -2.91201 | 0.000223 |
| **BMP2** | hsa-miR-410-3p | -0.30221 | 1.94E-12 | -3.89521 | 0.000272 |
| **BMP2** | hsa-let-7c-5p | -0.3012 | 2.32E-12 | -0.58871 | 0.067294 |
| **BMP2** | hsa-miR-374a-5p | -0.29037 | 1.46E-11 | -0.62756 | 0.019825 |
| **BMP2** | hsa-miR-329-3p | -0.27493 | 1.47E-10 | -3.83834 | 0.000166 |
| **BMP2** | hsa-miR-106b-5p | -0.2663 | 6.62E-10 | -0.34509 | 0.278938 |
| **BMP2** | hsa-miR-93-5p | -0.26149 | 1.36E-09 | 0.572642 | 0.029586 |
| **BMP2** | hsa-let-7g-5p | -0.25396 | 4.08E-09 | -1.5784 | 0.000178 |
| **BMP2** | hsa-let-7f-5p | -0.22948 | 1.15E-07 | -1.28294 | 0.003073 |
| **BMP2** | hsa-miR-488-3p | -0.22168 | 3.09E-07 | -1.94111 | 0.000198 |
| **BMP2** | hsa-miR-944 | -0.21316 | 8.26E-07 | -0.07818 | 0.166658 |
| **BMP2** | hsa-miR-362-3p | -0.18944 | 1.24E-05 | 0.051741 | 0.964066 |
| **BMP2** | hsa-miR-214-3p | -0.18372 | 2.28E-05 | -0.03736 | 0.343117 |
| **BMP2** | hsa-miR-140-5p | -0.18197 | 2.82E-05 | -0.94716 | 0.002177 |
| **BMP2** | hsa-miR-383-5p | -0.17032 | 8.79E-05 | -2.88461 | 0.000429 |
| **BMP2** | hsa-miR-20b-5p | -0.15153 | 0.000501 | -0.5735 | 0.06686 |
| **BMP2** | hsa-miR-106a-5p | -0.15134 | 0.000509 | -0.75224 | 0.008346 |
| **BMP2** | hsa-miR-129-5p | -0.1487 | 0.000639 | -4.21301 | 0.000174 |
| **BMP2** | hsa-miR-340-5p | -0.14475 | 0.00088 | -2.37712 | 0.000162 |
| **BMP2** | hsa-miR-374b-5p | -0.13823 | 0.001512 | -0.14679 | 0.552245 |
| **BMP2** | hsa-miR-4731-5p | -0.08432 | 0.053503 | -0.05492 | 0.146053 |
| **BMP2** | hsa-let-7b-5p | -0.07059 | 0.106189 | -0.18422 | 0.700145 |
| **BMP2** | hsa-miR-1277-5p | -0.06986 | 0.109877 | 0.00334 | 0.787456 |
| **BMP2** | hsa-let-7a-5p | -0.06689 | 0.1258 | -0.64455 | 0.028515 |
| **BMP2** | hsa-miR-3121-3p | -0.04487 | 0.304859 | 0.008597 | 0.653262 |
| **BMP2** | hsa-miR-526b-3p | -0.02838 | 0.516428 | 0.01101 | 0.749527 |
| **BMP2** | hsa-miR-17-5p | -0.02774 | 0.525747 | 0.0545 | 0.938604 |
| **BMP2** | hsa-miR-519d-3p | -0.00633 | 0.884956 | -0.16928 | 5.91E-05 |
| **BMP2** | hsa-miR-588 | 0.014558 | 0.739297 | 0.002665 | 0.801781 |
| **BMP2** | hsa-miR-520d-5p | 0.016342 | 0.708713 | 0.01301 | 0.706862 |
| **BMP2** | hsa-miR-1276 | 0.034288 | 0.433045 | -0.13871 | 0.310287 |
| **BMP2** | hsa-miR-3145-3p | 0.058768 | 0.178787 | 0.022419 | 0.402585 |
| **BMP2** | hsa-miR-524-5p | 0.0752 | 0.085182 | 0.017877 | 0.670058 |
| **BMP2** | hsa-miR-20a-5p | 0.157543 | 0.000295 | 0.276106 | 0.404991 |
| **BMP2** | hsa-miR-9-5p | 0.559272 | 0 | 0.784348 | 0.00069 |
